# Practical and Metal-Free Synthesis of Novel Enantiopure Amides Containing the Potentially Bioactive 5-Nitroimidazole Moiety

**DOI:** 10.3390/molecules21111472

**Published:** 2016-11-04

**Authors:** Cédric Spitz, Fanny Mathias, Alain Gamal Giuglio-Tonolo, Thierry Terme, Patrice Vanelle

**Affiliations:** Aix-Marseille Université, CNRS, ICR UMR 7273, Equipe Pharmaco-Chimie Radicalaire, Faculté de Pharmacie, 27 Boulevard Jean Moulin, CS 30064, 13385 Marseille CEDEX 05, France; cedric.spitz@univ-amu.fr (C.S.); fanny.mathias@laposte.net (F.M.); gamal.giuglio-tonolo@univ-amu.fr (A.G.G.-T.); thierry.terme@univ-amu.fr (T.T.)

**Keywords:** 5-nitroimidazoles, metal-free, amides

## Abstract

We report here a practical and metal-free synthesis of novel enantiopure amides containing the drug-like 5-nitroimidazole scaffold. The first step was a metal-free diastereoselective addition of 4-(4-(chloromethyl)phenyl)-1,2-dimethyl-5-nitro-1*H*-imidazole to enantiomerically pure *N*-*tert*-butanesulfinimine. Then, the *N*-*tert*-butanesulfinyl–protected amine was easily deprotected under acidic conditions. Finally, the primary amine was coupled with different acid chlorides or acids to give the corresponding amides. The mild reaction conditions and high tolerance for various substitutions make this approach attractive for constructing pharmacologically interesting 5-nitroimidazoles.

## 1. Introduction

**Figure 1:**
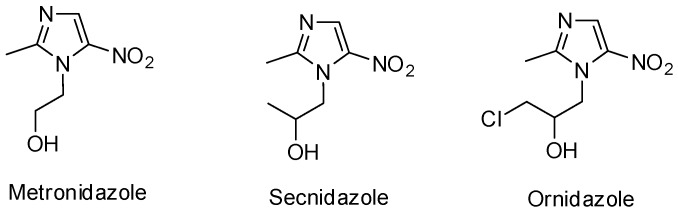
Structures of bioactive 5-nitroimidazoles.

The 5-nitroimidazole moiety is well known to exhibit a wide spectrum of anti-infectious activity [1,2,3,4,5,6]. Several 5-nitroimidazole–containing active principles such as metronidazole, secnidazole and ornidazole are commonly used in medicine (Figure 1). These chemotherapeutic agents inhibit the growth of both anaerobic bacteria and some anaerobic protozoa [7]. However, the 5-nitroimidazoles have been found to possess a high mutagenic activity in prokaryotic micro-organisms. So, a nitroimidazole possessing good pharmacological activities with no mutagenicity [8] would be of great interest.

However, the difficulty of generating organometallic nitro compounds significantly reduces the number of available methods for easily constructing various substituted 5-nitroimidazoles. In particular, the stereoselective addition of organometallics to enantiomerically pure imines, which is one of the most commonly used methods for the asymmetric synthesis of amines, appeared to be difficult to apply with a 5-nitroimidazole scaffold. So, an alternative approach could be the use of tetrakis(dimethylamino)ethylene (TDAE), an organic reducing agent which reacts with halogenated derivatives to generate a carbanion under mild conditions [9]. Based on this property, we recently developed a metal-free diastereoselective addition of 4-(4-(chloromethyl)phenyl)-1,2-dimethyl-5-nitro-1*H*-imidazole to several enantiopure *N*-*tert*-butanesulfinimines using TDAE [10].

Here, we wanted to use these mild conditions in order to synthesize *N*-*tert*-butanesulfinyl–protected sulfinylamine **3** on a gram scale with good diastereoselectivity. Then, the amine **3** can be easily deprotected under acidic conditions. Finally, as part of our research program for new bioactive compounds [11,12,13,14], the primary amine **4** was coupled with different acid chlorides or acids to give the corresponding amides **6**.

## 2. Results and Discussion

**Scheme 1:**
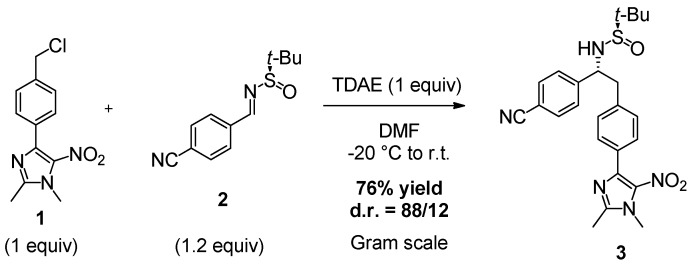
Diastereoselective addition of 4-(4-(chloromethyl)phenyl)-1,2-dimethyl-5-nitro-1*H*-imidazole (**1**) to *N*-*tert*-butanesulfinimine **2**.

First, the reaction between 4-(4-(chloromethyl)phenyl)-1,2-dimethyl-5-nitro-1*H*-imidazole (**1**) and enantiopure aromatic *N*-sulfinimine **2** in the presence of TDAE at −20 °C for 1 h, followed by 16 h at room temperature, led to the corresponding amine **3** in 76% yield and with a good 88/12 diastereoselectivity as shown in Scheme 1.

Interestingly, the diastereoselective addition of a 5-nitroimidazole benzyl chloride derivative to enantiopure sulfinimine can be done without metal and under mild conditions, tolerating various functional groups. In particular, the 4-cyano aromatic imine **2** was chosen not only because it is well tolerated under these metal-free conditions, but also because further structural modifications could be envisaged using the cyano moiety reactivity.

**Scheme 2:**
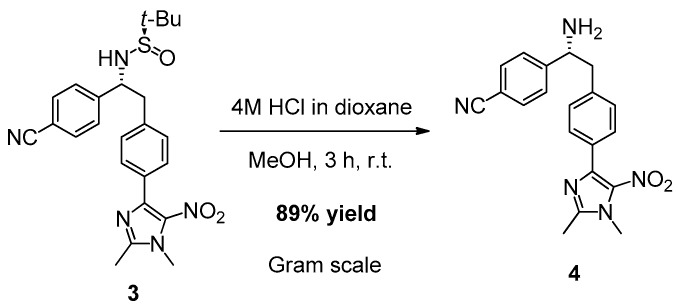
Removal of *tert*-butanesulfinyl group.

Besides the mild reaction conditions and the simplicity of the purification procedure, another advantage associated with this method is that it produces *N*-*tert*-butanesulfinyl–protected amines. Such a protecting group is easy to cleave under acidic conditions [15]. So, the *N*-*tert*-butanesulfinyl–protected amine **3** was deprotected under acidic conditions to give the corresponding free amine **4** in a very good 89% yield (Scheme 2).

Finally, in order to synthesize peptide-like molecules, we decided to prepare different substituted amides **6** from primary amine **4**. In this context, the reaction between primary amine **4** and several acid chlorides **5a**–**i** in the presence of triethylamine allowed the formation of amides **6a**–**i** in good yields (Table 1, Entries 1–9). Alternatively, in the presence of hydroxybenzotriazole (HOBt) and 1-ethyl-3-(3-dimethylaminopropyl)carbodiimide hydrochloride (EDC.HCl), free amine **4** can also be directly converted into amides **6j**–**l** from acids **5j**–**l** (see supporting information), instead of from more toxic and reactive acid chlorides (Entries 10–12).

Thus, several new benzamides, **6a**–**d** and **6j**, were synthesized in good yields (Entries 1–4, and 10). Under these conditions, the heteroaromatic acid chloride **5i** also allowed the formation of the corresponding amide **6i** in good yield (Entry 9). Alkyl substitutions on the acid chloride or acid derivatives also produced good yields of amides **6e**, **6h** and **6k** (Entries 5, 8 and 11). Interestingly, trihalogenated substitutions on the acid chlorides (Entries 6–7) and the presence of a double bond on the acid (Entry 12) were all well tolerated.

## 3. Experimental Section

### 3.1. General

Melting points were determined on a Büchi melting point B-540 apparatus (BÜCHI Labortechnik AG, Flawil, Switzerland) and are uncorrected. Element analyses were performed on a Thermo Finnigan EA1112 (San Jose, CA, USA) at the spectropole of the Aix-Marseille University. Both ^1^H- and ^13^C-NMR spectra were determined on a Bruker AC 250 spectrometer (Wissembourg, France) at the Service de RMN de la Faculté de Pharmacie de Marseille of the Aix-Marseille University. The ^1^H and the ^13^C chemical shifts are reported from CDCl_3_ peaks: ^1^H (7.26 ppm) and ^13^C (77.16 ppm). Multiplicities are represented by the following notations: s, singlet; d, doublet; t, triplet; q, quartet; m, a more complex multiplet or overlapping multiplets. The following adsorbents were used for column chromatography: Silica gel 60 (Merck, Darmstadt, Germany, particle size 0.063–0.200 mm, 70–230 mesh ASTM). TLC was performed on 5 cm × 10 cm aluminium plates coated with silica gel 60 F_254_ (Merck) in an appropriate solvent. Purity of synthetized compounds was checked with LC-MS analyses which were realized at the Faculté de Pharmacie de Marseille with a Thermo Scientific Accela High Speed LC System^®^ coupled with a single quadrupole mass spectrometer Thermo MSQ Plus^®^ (San Jose, CA, USA). The RP-HPLC column used is a Thermo Hypersil Gold^®^ 50 × 2.1 mm (C18 bounded), with particles of 1.9 µm diameter. The volume of sample injected on the column was 1 µL. The chromatographic analysis, total duration of 8 min, is made with the gradient of following solvents: *t* = 0 min, water:ethanol 50:50; 0 < *t* < 4 min, linear increase in the proportion of water to a ratio water/methanol 95:5; 4 < *t* < 6 min, water/methanol 95:5; 6 < *t* < 7 min, linear decrease in the proportion of water to return to a ratio 50:50 water/methanol; 6 < *t* < 7 min, water/methanol 50:50. The water used was buffered with 5 mM ammonium acetate. The retention times (*t*_R_) of the molecules analyzed are indicated in min.

Compound **1** was synthesized according to a previously described procedure [16]. Compound **2** was synthesized according to a previously described procedure from 4-cyanobenzaldehyde and enantiopure (*R*)-(+)-*tert*-butanesulfinamide [17].

### 3.2. Synthesis of (R)-N-((R)-1-(4-Cyanophenyl)-2-(4-(1,2-dimethyl-5-nitro-1H-imidazol-4-yl)phenyl)ethyl)-2-methylpropane-2-sulfinamide *(**3**)*

Compound **3** was synthesized on a gram scale using a previously described small-scale procedure: To a stirred solution of *N*-sulfinimine **2** (1.15 g, 4.91 mmol, 1.2 equiv) in anhydrous DMF (15 mL) at −20 °C was added TDAE (0.952 mL, 4.09 mmol, 1 equiv) followed by dropwise addition of a solution of 4-(4-(chloromethyl)phenyl)-1,2-dimethyl-5-nitro-1*H*-imidazole (**1**) (1.09 g, 4.09 mmol, 1 equiv) in anhydrous DMF (15 mL). The solution was vigorously stirred at −20 °C for 1 h and then maintained at room temperature for 16 h. Water (30 mL) was added and the aqueous solution was extracted with CH_2_Cl_2_ (3 × 30 mL). The combined organic layer was washed with H_2_O (50 mL) and dried over MgSO_4_. Evaporation of the solvent furnished the crude product. Purification by silica gel chromatography afforded **3** as a yellow solid (1.44 g, 3.11 mmol, 76%). Experimental data are in accordance with literature [10].

### 3.3. Synthesis of (R)-4-(1-Amino-2-(4-(1,2-dimethyl-5-nitro-1H-imidazol-4-yl)phenyl)ethyl)benzonitrile *(**4**)*

To a stirred solution of *N*-protected amine **3** (1.34 g, 2.88 mmol, 1 equiv) in MeOH (80 mL) was added 4 M HCl in dioxane (4.31 mL, 17.2 mmol, 6 equiv) and the solution was stirred at room temperature for 3 h. The reaction mixture was then concentrated under reduced pressure, diluted with water (30 mL) and HCl_conc._ was added until pH = 1. The mixture was washed with EtOAc (2 × 30 mL). Then, a saturated NaHCO_3_ solution was added to the aqueous layer until pH = 7–8. The aqueous layer was extracted with EtOAc (3 × 30 mL). The combined organic layers were dried over MgSO_4_, filtered, and concentrated under reduced pressure to allow pure primary amine **4** as a yellow oil (0.93 g, 2.57 mmol, 89%). ${\left[\alpha \right]}_{D}^{25}$ = −29.5° (*c* = 0.1, CH_3_OH). ^1^H-NMR (250 MHz, CDCl_3_) δ 7.71–7.59 (m, 4H), 7.46 (d, *J* = 8.3 Hz, 2H), 7.19 (d, *J* = 7.8 Hz, 2H), 4.32–4.27 (m, 1H), 3.91 (s, 3H), 3.02–2.81 (m, 2H), 2.52 (s, 3H), 1.69 (*br*-s, 2H). ^13^C NMR (63 MHz, CD_3_OD) δ 150.3, 143.3, 140.7, 136.1, 133.3, 131.5, 130.7, 130.3, 130.1, 129.0, 119.7, 111.9, 58.6, 46.3, 34.6, 13.6. HRMS (ESI): *m*/*z* [M + H]^+^ calcd for [C_20_H_20_N_5_O_2_]^+^: 362.1612; found: 362.1611.

### 3.4. General Procedure for the Synthesis of Amides ***6a**–**i***

To a stirred solution of primary amine **4** (50 mg, 0.14 mmol, 1 equiv) in DCM (1 mL) was added the relevant acid chloride **5a**–**i** (0.16 mmol, 1.15 equiv) and triethylamine (20 μL, 0.14 mmol, 1 equiv). The reaction mixture was stirred at room temperature for 16 h. DCM (10 mL) and water (10 mL) were added and the layers were separated. The aqueous layer was extracted with DCM (2 × 10 mL) and the combined organic layers were dried over MgSO_4_ and filtered. Evaporation of the solvent furnished the crude product. Purification by silica gel chromatography (EtOAc/MeOH: From 10/0 to 9/1 depending on the polarity of substrates) allowed pure amide products **6a**–**i**.

*(R)-N-(1-(4-Cyanophenyl)-2-(4-(1,2-dimethyl-5-nitro-1H-imidazol-4-yl)phenyl)ethyl)benzamide* (**6a**). Yellow solid, m.p. 194–196 °C. ^1^H-NMR (250 MHz, CDCl_3_) δ 7.68 (d, *J* = 8.3 Hz, 4H), 7.60 (d, *J* = 8.3 Hz, 2H), 7.50–7.36 (m, 5H), 7.13 (d, *J* = 8.0 Hz, 2H), 6.55 (d, *J* = 7.2 Hz, 1H), 5.49 (dd, *J* = 7.2, 6.9 Hz, 1H), 3.90 (s, 3H), 3.24 (d, *J* = 6.9 Hz, 2H), 2.51 (s, 3H). ^13^C-NMR (63 MHz, CDCl_3_) δ 167.1, 148.4, 147.3, 143.0, 137.7, 134.3, 132.6, 131.9, 131.1, 130.1, 129.1, 128.8, 127.6, 127.1, 118.7, 111.6, 54.7, 42.3, 34.1, 14.1 (CNO_2_ not visible under these conditions). HRMS (ESI): *m*/*z* [M + H]^+^ calcd for [C_27_H_24_N_5_O_3_]^+^: 466.1874; found: 466.1873. 

*(R)-N-(1-(4-Cyanophenyl)-2-(4-(1,2-dimethyl-5-nitro-1H-imidazol-4-yl)phenyl)ethyl)-4-nitrobenzamide* (**6b**). Yellow solid, m.p. 182–184 °C. ^1^H-NMR (250 MHz, CDCl_3_) δ 8.25 (d, *J* = 8.8 Hz, 2H), 7.79 (d, *J* = 8.9 Hz, 2H), 7.70 (d, *J* = 8.2 Hz, 2H), 7.64 (d, *J* = 8.4 Hz, 2H), 7.41 (d, *J* = 8.2 Hz, 2H), 7.17 (d, *J* = 8.2 Hz, 2H), 6.53 (d, *J* = 7.0 Hz, 1H), 5.49 (dd, *J* = 7.0, 6.8 Hz, 1H), 3.92 (s, 3H), 3.36–3.18 (m, 2H), 2.53 (s, 3H). ^13^C-NMR (63 MHz, CDCl_3_) δ 165.3, 150.1, 148.5, 146.7, 143.0, 139.8, 137.4, 132.7, 131.4, 130.3, 129.0, 128.3, 127.6, 124.1, 118.6, 112.0, 55.1, 42.2, 34.2, 14.2 (CNO_2_ not visible under these conditions). HRMS (ESI): *m*/*z* [M + H]^+^ calcd for [C_27_H_23_N_6_O_5_]^+^: 511.1724; found: 511.1725. 

*(R)-4-Chloro-N-(1-(4-cyanophenyl)-2-(4-(1,2-dimethyl-5-nitro-1H-imidazol-4-yl)phenyl)ethyl)benzamide* (**6c**). Yellow solid, m.p. 173–174 °C. ^1^H-NMR (250 MHz, CDCl_3_) δ 7.67 (d, *J* = 8.0 Hz, 2H), 7.59 (d, *J* = 8.5 Hz, 4H), 7.38–7.34 (m, 4H), 7.12 (d, *J* = 8.0 Hz, 2H), 6.61 (d, *J* = 7.1 Hz, 1H), 5.45 (dd, *J* = 7.1, 6.8 Hz, 1H), 3.90 (s, 3H), 3.22 (d, *J* = 6.8 Hz, 2H), 2.50 (s, 3H). ^13^C-NMR (63 MHz, CDCl_3_) δ 166.1, 148.4, 147.1, 143.0, 138.3, 137.6, 132.6, 131.2, 130.2, 129.2, 129.1, 128.5, 127.6, 118.6, 111.8, 54.8, 42.3, 34.2, 14.2 (CNO_2_ not visible under these conditions and 1 carbon signal missing due to overlap). HRMS (ESI): *m*/*z* [M + H]^+^ calcd for [C_27_H_23_N_5_O_3_Cl]^+^: 500.1484; found: 500.1483.

*(R)-2-Bromo-N-(1-(4-cyanophenyl)-2-(4-(1,2-dimethyl-5-nitro-1H-imidazol-4-yl)phenyl)ethyl)benzamide* (**6d**). Yellow solid, m.p. 196–198 °C. ^1^H-NMR (250 MHz, CDCl_3_) δ 7.67 (d, *J* = 8.2 Hz, 2H), 7.60 (d, *J* = 8.5 Hz, 2H), 7.53 (d, *J* = 8.0 Hz, 1H), 7.42 (d, *J* = 8.2 Hz, 2H), 7.34–7.30 (m, 2H), 7.27–7.24 (m, 1H), 7.14 (d, *J* = 8.2 Hz, 2H), 6.59 (d, *J* = 7.2 Hz, 1H), 5.50 (dd, *J* = 7.2, 6.5 Hz, 1H), 3.89 (s, 3H), 3.23–3.18 (m, 2H), 2.50 (s, 3H). ^13^C-NMR (63 MHz, CDCl_3_) δ 167.0, 148.4, 146.8, 143.0, 137.6, 137.3, 133.6, 132.6, 131.6, 131.1, 130.1, 129.2, 127.84, 127.75, 119.3, 118.7, 111.8, 55.1, 42.2, 34.1, 14.1 (CNO_2_ not visible under these conditions and 1 carbon signal missing due to overlap). HRMS (ESI): *m*/*z* [M + H]^+^ calcd for [C_27_H_23_N_5_O_3_Br]^+^: 546.0963; found: 546.0964. 

*(R)-N-(1-(4-Cyanophenyl)-2-(4-(1,2-dimethyl-5-nitro-1H-imidazol-4-yl)phenyl)ethyl)acetamide* (**6e**). Yellow solid, m.p. 114–116 °C. ^1^H-NMR (250 MHz, CDCl_3_) δ 7.65 (d, *J* = 8.0 Hz, 2H), 7.59 (d, *J* = 8.0 Hz, 2H), 7.31 (d, *J* = 8.0 Hz, 2H), 7.08 (d, *J* = 8.0 Hz, 2H), 5.90 (d, *J* = 7.8 Hz, 1H), 5.32 (dd, *J* = 7.8, 6.8 Hz, 1H), 3.92 (s, 3H), 3.23 (d, *J* = 6.8 Hz, 2H), 2.54 (s, 3H), 1.96 (s, 3H). ^13^C-NMR (63 MHz, CDCl_3_) δ 169.6, 148.4, 147.4, 143.0, 137.9, 132.5, 131.0, 130.0, 129.0, 127.6, 118.6, 111.7, 54.3, 42.2, 34.1, 23.2, 14.1 (CNO_2_ not visible under these conditions). HRMS (ESI): *m*/*z* [M + H]^+^ calcd for [C_22_H_22_N_5_O_3_]^+^: 404.1717; found: 404.1720. 

*(R)-2,2,2-Trichloro-N-(1-(4-cyanophenyl)-2-(4-(1,2-dimethyl-5-nitro-1H-imidazol-4-yl)phenyl)ethyl)acetamide* (**6f**). Yellow solid, m.p. 109–111 °C. ^1^H-NMR (250 MHz, CDCl_3_) δ 7.67 (d, *J* = 8.3 Hz, 2H), 7.63 (d, *J* = 8.5 Hz, 2H), 7.33 (d, *J* = 8.3 Hz, 2H), 7.09 (d, *J* = 8.3 Hz, 3H), 5.24 (dd, *J* = 7.2, 7.0 Hz, 1H), 3.90 (s, 3H), 3.23 (dd, *J* = 7.0, 3.0 Hz, 2H), 2.51 (s, 3H). ^13^C-NMR (63 MHz, CDCl_3_) δ 161.4, 148.4, 145.3, 142.7, 136.6, 132.8, 131.5, 130.3, 129.1, 127.4, 118.4, 112.5, 92.7, 56.2, 41.9, 34.1, 14.1 (CNO_2_ not visible under these conditions). HRMS (ESI): *m*/*z* [M + H]^+^ calcd for [C_22_H_19_N_5_O_3_Cl_3_]^+^: 506.0548; found: 506.0546. 

*(R)-2,2,2-Tribromo-N-(1-(4-cyanophenyl)-2-(4-(1,2-dimethyl-5-nitro-1H-imidazol-4-yl)phenyl)ethyl)acetamide* (**6g**). Yellow solid, m.p. 100–102 °C. ^1^H-NMR (250 MHz, CDCl_3_) δ 7.69 (d, *J* = 8.3 Hz, 2H), 7.65 (d, *J* = 8.5 Hz, 2H), 7.36 (d, *J* = 8.3 Hz, 2H), 7.13 (d, *J* = 8.3 Hz, 3H), 5.26 (dd, *J* = 7.2, 7.0 Hz, 1H), 3.92 (s, 3H), 3.34–3.14 (m, 2H), 2.54 (s, 3H). ^13^C-NMR (63 MHz, CDCl_3_) δ 161.7, 148.5, 145.4, 142.9, 136.5, 134.9, 132.7, 131.2, 130.2, 129.1, 127.3, 118.6, 111.9, 56.3, 41.8, 35.7, 34.4, 14.3. HRMS (ESI): *m*/*z* [M + H]^+^ calcd for [C_22_H_19_N_5_O_3_Br_3_]^+^: 639.9013; found: 639.9011.

*(R)-N-(1-(4-Cyanophenyl)-2-(4-(1,2-dimethyl-5-nitro-1H-imidazol-4-yl)phenyl)ethyl)palmitamide* (**6h**). Yellow solid, m.p. 153–155 °C. ^1^H-NMR (250 MHz, CDCl_3_) δ 7.68–7.58 (m, 4H), 7.33–7.26 (m, 2H), 7.08 (d, *J* = 8.0 Hz, 2H), 5.83 (d, *J* = 7.0 Hz, 1H), 5.38–5.29 (m, 1H), 3.92 (s, 3H), 3.12 (d, *J* = 6.5 Hz, 2H), 2.53 (s, 3H), 2.15 (t, *J* = 7.5 Hz, 2H), 1.30–1.20 (m, 26H), 0.89–0.87 (m, 3H). ^13^C-NMR (63 MHz, CDCl_3_) δ 172.7, 148.4, 147.4, 143.1, 137.7, 132.5, 131.1, 130.0, 129.0, 127.6, 118.7, 111.7, 54.1, 42.3, 36.8, 34.1, 32.1, 29.83, 29.79, 29.76, 29.6, 29.48, 29.45, 29.40, 25.7, 22.8, 14.2 (CNO_2_ not visible under these conditions). HRMS (ESI): *m*/*z* [M + H]^+^ calcd for [C_36_H_50_N_5_O_3_]^+^: 600.3908; found: 600.3906.

*(R)-N-(1-(4-Cyanophenyl)-2-(4-(1,2-dimethyl-5-nitro-1H-imidazol-4-yl)phenyl)ethyl)furan-2-carboxamide* (**6i**). Yellow solid, m.p. 191–193 °C. ^1^H-NMR (250 MHz, CDCl_3_) δ 7.66 (d, *J* = 8.3 Hz, 2H), 7.59 (d, *J* = 8.3 Hz, 2H), 7.43–7.35 (m, 3H), 7.13–7.06 (m, 3H), 6.77 (d, *J* = 7.3 Hz, 1H), 6.49–6.46 (m, 1H), 5.50–5.42 (m, 1H), 3.89 (s, 3H), 3.23 (d, *J* = 7.0 Hz, 2H), 2.50 (s, 3H). ^13^C-NMR (63 MHz, CDCl_3_) δ 157.9, 148.4, 148.0, 147.0, 144.3, 143.1, 137.5, 132.6, 131.2, 130.0, 129.1, 127.7, 118.6, 114.9, 112.4, 111.9, 54.1, 42.4, 34.1, 14.1 (CNO_2_ not visible under these conditions). HRMS (ESI): *m*/*z* [M + H]^+^ calcd for [C_25_H_22_N_5_O_4_]^+^: 456.1666; found: 456.1664. 

### 3.5. General Procedure for the Synthesis of Amides ***6j**–**l***

The relevant acid **5j**–**l** (0.11 mmol, 1.1 equiv), HOBt (20 mg, 0.13 mmol, 1.3 equiv) and EDC.HCl (25 mg, 0.13 mmol, 1.3 equiv) were stirred in DMF (0.5 mL) for 15 min. Then, primary amine **4** (36 mg, 0.1 mmol, 1 equiv) was added and the reaction mixture was stirred at room temperature for 16 h. EtOAC (10 mL) and water (10 mL) were added and the layers were separated. The aqueous layer was extracted with EtOAc (2 × 10 mL) and the combined organic layers were washed with water (2 × 10 mL), dried over MgSO_4_ and filtered. Evaporation of the solvent furnished the crude product. Purification by silica gel chromatography (EtOAc/MeOH: from 10/0 to 9/1 depending on the polarity of substrates) allowed pure amide products **6j**–**l**.

*(R)-3-Chloro-N-(1-(4-cyanophenyl)-2-(4-(1,2-dimethyl-5-nitro-1H-imidazol-4-yl)phenyl)ethyl)benzamide* (**6j**). Yellow solid, m.p. 135–137 °C. ^1^H-NMR (250 MHz, CDCl_3_) δ 7.68–7.65 (m, 3H), 7.59 (d, *J* = 8.0 Hz, 2H), 7.53–7.43 (m, 2H), 7.39–7.30 (m, 3H), 7.13 (d, *J* = 8.0 Hz, 2H), 6.64 (d, *J* = 7.0 Hz, 1H), 5.48–5.43 (m, 1H), 3.90 (s, 3H), 3.22 (d, *J* = 6.8 Hz, 2H), 2.50 (s, 3H). ^13^C-NMR (63 MHz, CDCl_3_) δ 165.8, 148.5, 146.9, 142.8, 137.7, 135.7, 134.9, 134.8, 132.6, 132.0, 130.6, 130.14, 130.08, 129.0, 127.5, 125.1, 118.8, 111.5, 54.8, 42.1, 34.4, 14.2 (CNO_2_ not visible under these conditions). HRMS (ESI): *m*/*z* [M + H]^+^ calcd for [C_27_H_23_N_5_O_3_Cl]^+^: 500.1484; found: 500.1485.

*(R)-N-(1-(4-Cyanophenyl)-2-(4-(1,2-dimethyl-5-nitro-1H-imidazol-4-yl)phenyl)ethyl)-2-phenylacetamide* (**6k**). Yellow solid, m.p. 174–176 °C. ^1^H-NMR (250 MHz, CDCl_3_) δ 7.61–7.54 (m, 4H), 7.37–7.34 (m, 3H), 7.19–7.13 (m, 4H), 6.85 (d, *J* = 8.0 Hz, 2H), 5.80 (d, *J* = 7.3 Hz, 1H), 5.26 (dd, *J* = 7.3, 6.8 Hz, 1H), 3.92 (s, 3H), 3.53 (s, 2H), 3.03 (dd, *J* = 14.0, 6.2 Hz, 1H), 2.88 (dd, *J* = 14.0, 7.5 Hz, 1H), 2.54 (s, 3H). ^13^C-NMR (63 MHz, CDCl_3_) δ 170.6, 148.5, 147.1, 142.9, 137.3, 134.9, 134.5, 132.5, 130.5, 130.0, 129.6, 129.4, 128.9, 127.8, 127.2, 118.8, 111.3, 54.0, 43.8, 42.1, 34.4, 14.3. HRMS (ESI): *m*/*z* [M + H]^+^ calcd for [C_28_H_26_N_5_O_3_]^+^: 480.2030; found: 480.2029.

*(R,E)-3-(Benzo[d][1,3]dioxol-5-yl)-N-(1-(4-cyanophenyl)-2-(4-(1,2-dimethyl-5-nitro-1H-imidazol-4-yl)phenyl)ethyl)acrylamide* (**6l**). Yellow solid, m.p. 123–125 °C. ^1^H-NMR (250 MHz, CDCl_3_) δ 7.65 (d, *J* = 8.3 Hz, 2H), 7.57 (d, *J* = 8.3 Hz, 2H), 7.46 (d, *J* = 15.5 Hz, 1H), 7.34 (d, *J* = 8.3 Hz, 2H), 7.09 (d, *J* = 8.0 Hz, 2H), 6.92 (d, *J* = 8.3 Hz, 2H), 6.75 (d, *J* = 8.0 Hz, 1H), 6.28–6.20 (m, 2H), 5.97 (s, 2H), 5.46–5.37 (m, 1H), 3.90 (s, 3H), 3.16 (d, *J* = 7.0 Hz, 2H), 2.51 (s, 3H). ^13^C-NMR (63 MHz, CDCl_3_) δ 165.8, 149.4, 148.5, 148.3, 147.3, 143.0, 141.9, 137.8, 134.8, 132.5, 130.5, 130.0, 129.1, 129.0, 127.5, 124.3, 118.9, 117.9, 111.2, 108.6, 106.4, 101.6, 54.3, 42.1, 34.4, 14.2. HRMS (ESI): *m*/*z* [M + H]^+^ calcd for [C_30_H_26_N_5_O_5_]^+^: 536.1928; found: 536.1926.

## 4. Conclusions

In conclusion, the metal-free diastereoselective addition of 4-(4-(chloromethyl)phenyl)-1,2-dimethyl-5-nitro-1*H*-imidazole (**1**) to enantiomerically pure *N*-*tert*-butanesulfinimine **2** allowed the synthesis of *N*-*tert*-butanesulfinyl–protected amine **3** in good yield and with good diastereoselectivity. Then, amine **3** was easily deprotected under acidic conditions to give primary amine **4** which was coupled with different acid chlorides or acids to give the corresponding amides **6** in good yields. The mild reaction conditions for all steps and the high tolerance for various substitutions make this approach attractive for constructing pharmacologically interesting 5-nitroimidazoles. The pharmacological evaluation of all synthesized compounds is under active investigation.

## Figures and Tables

**Table 1:**
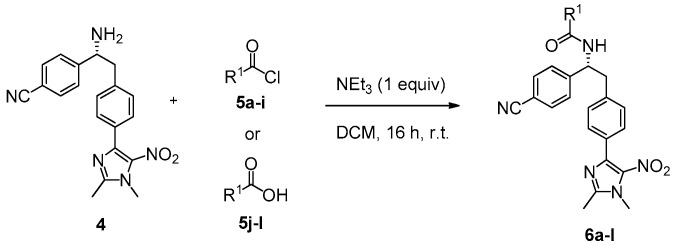
Synthesis of amides **6a**–**l** from primary amine **4** ^a^.

Entry	R^1^	Yield of 6 (%) ^b^
1	C_6_H_5_	75 (**6a**)
2	4-NO_2_C_6_H_4_	83 (**6b**)
3	4-ClC_6_H_4_	80 (**6c**)
4	2-BrC_6_H_4_	64 (**6d**)
5	CH_3_	79 (**6e**)
6	CCl_3_	74 (**6f**)
7	CBr_3_	73 (**6g**)
8	CH_3_(CH_2_)_14_	69 (**6h**)
9	2-Furyl	68 (**6i**)
10 ^c^	3-ClC_6_H_4_	70 (**6j**)
11 ^c^	C_6_H_5_CH_2_	63 (**6k**)
12 ^c^	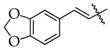	73 (**6l**)

^a^ Reactions were performed using 1 equiv of amine **4**, 1.15 equiv of acid chlorides **5a**–**i** and 1 equiv of triethylamine in DCM at room temperature for 16 h; ^b^ Yields of pure amides **6** after purification by chromatography; ^c^ Reactions were performed using 1 equiv of amine **4**, 1.1 equiv of acids **5j**–**l**, 1.3 equiv of HOBt and 1.3 equiv of EDC.HCl in DMF at room temperature for 16 h.

## Supplementary Materials

Supplementary materials can be accessed at: http://www.mdpi.com/1420-3049/21/11/1472/s1.
