# Supplementary material for: Practical and Metal-Free Synthesis of Novel Enantiopure Amides Containing the Potentially Bioactive 5-Nitroimidazole Moiety
Source: Molecules. 2016 Nov 4;21(11):1472. doi: 10.3390/molecules21111472 (PMC6273685; doi:10.3390/molecules21111472)
Supplement: Supplementary file 1 [file molecules-21-01472-s001.pdf]

Supplementary Materials: Practical and Metal-Free Synthesis of Novel Enantiopure Amides Containing the Potentially Bioactive 5-Nitroimidazole Moiety

Cédric Spitz, Fanny Mathias, Alain Gamal Giuglio-Tonolo, Thierry Terme and Patrice Vanelle

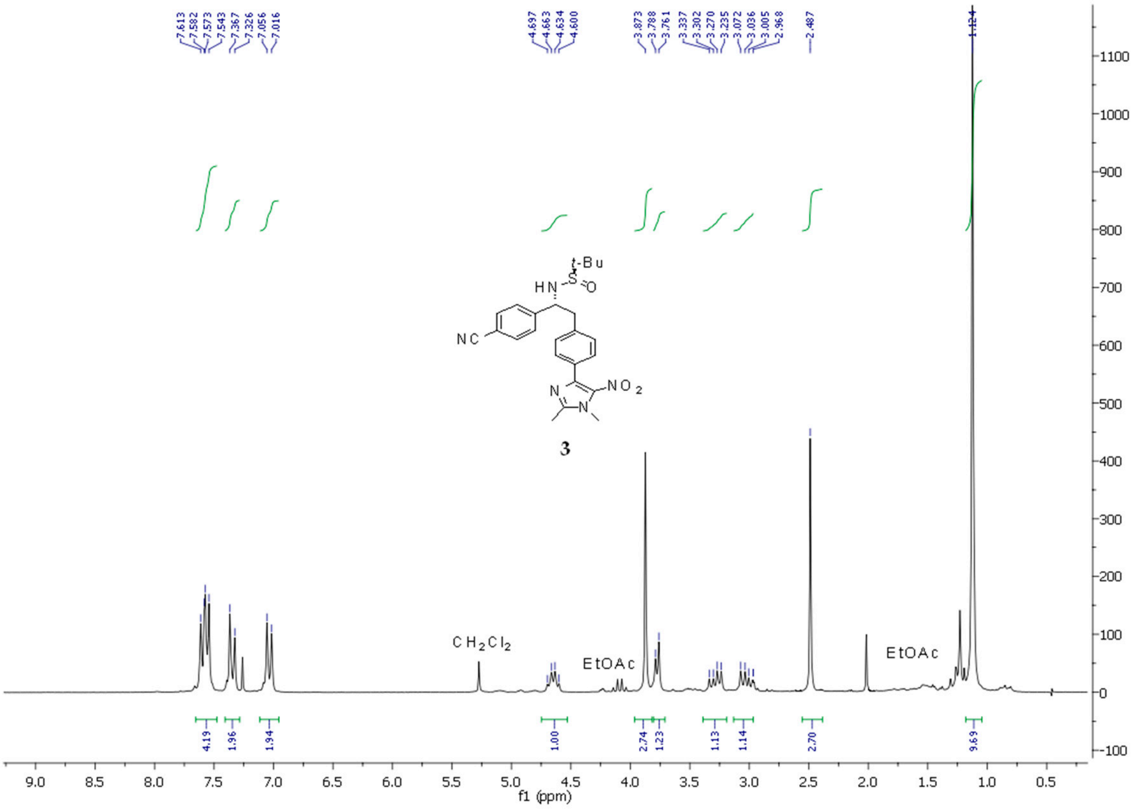

Figure S1. ^{1}H spectra of **3**.

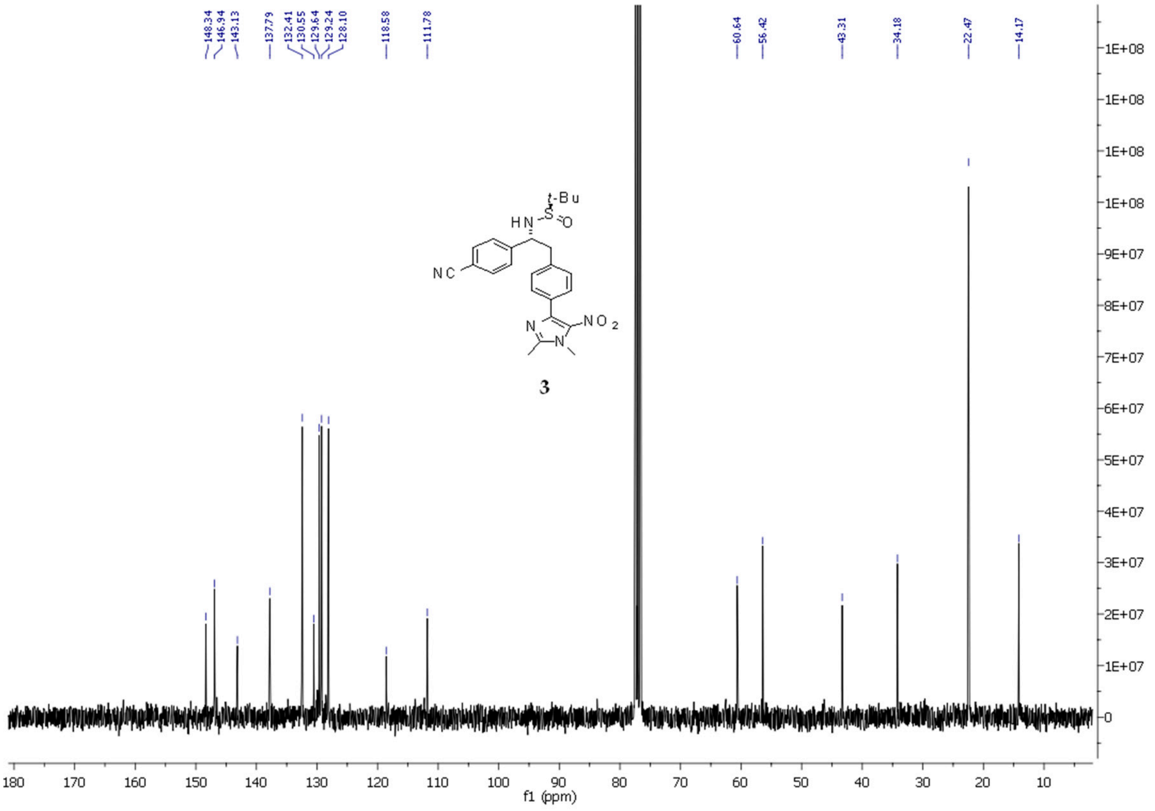

Figure S2. ^{13}C-NMR spectra of **3**.

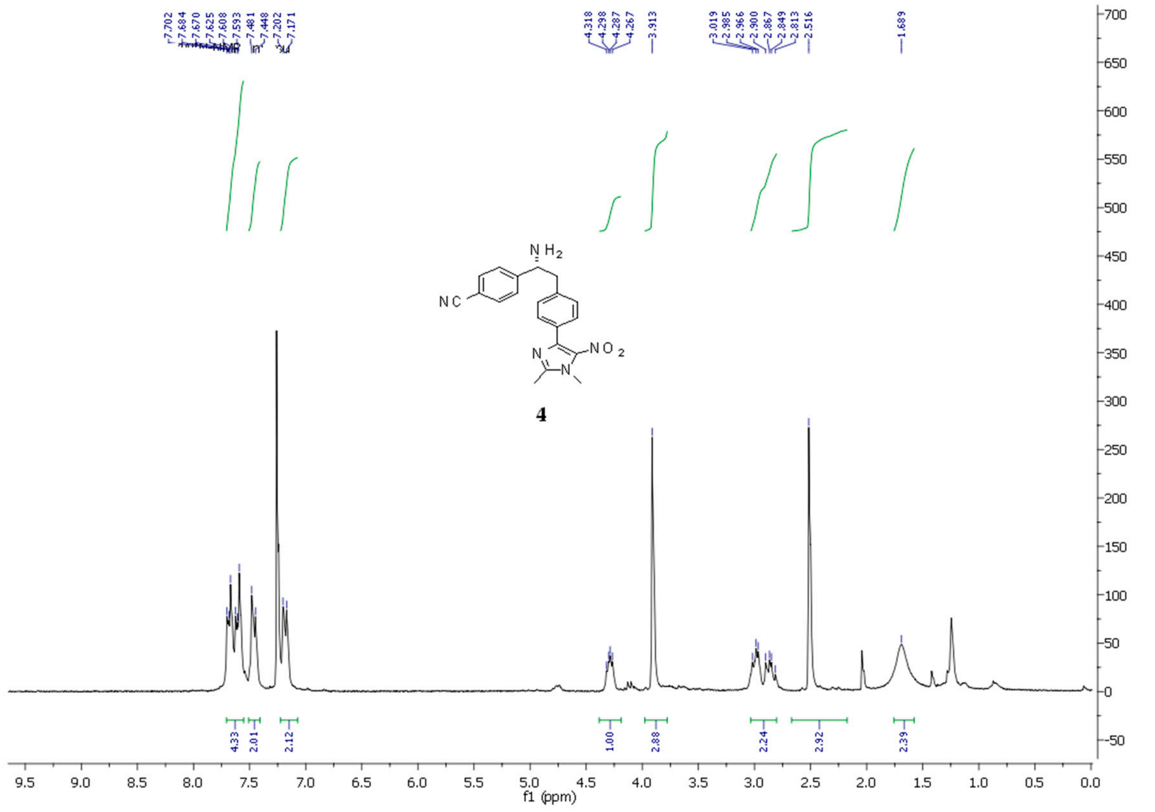

Figure S3. ^{1}H-NMR spectra of **4**.

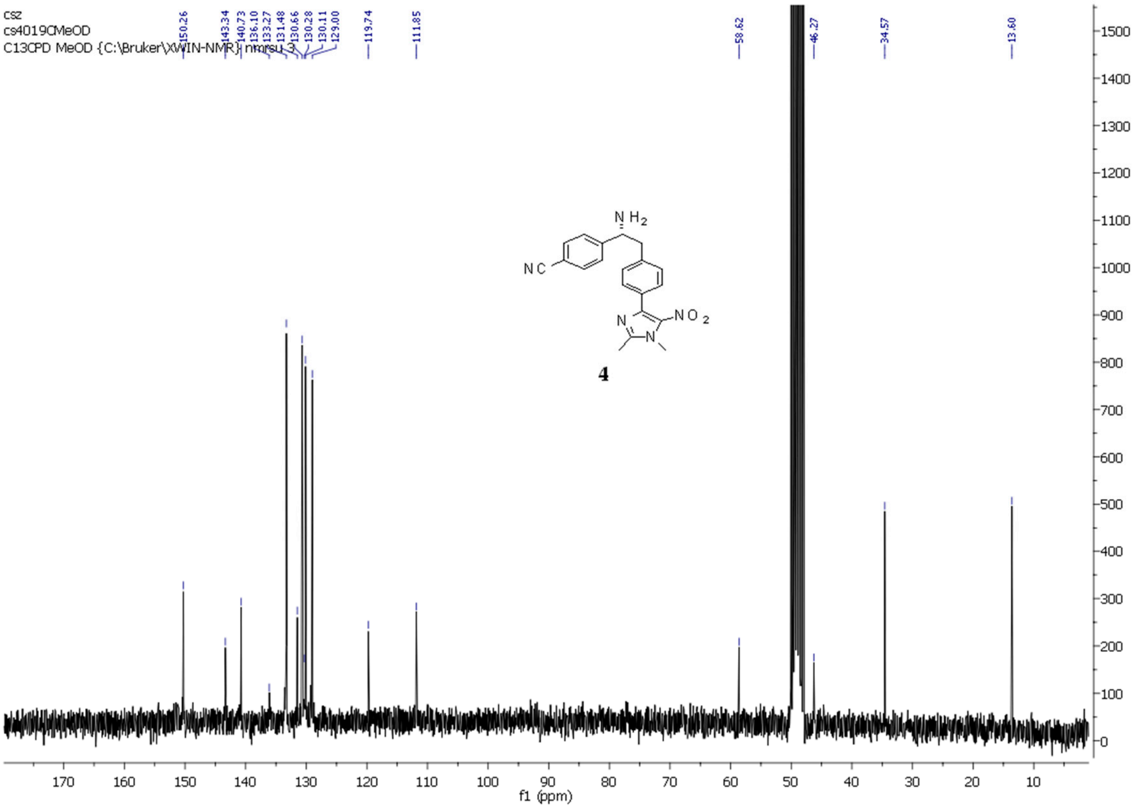

Figure S4. ^{13}C-NMR spectra of **4**.

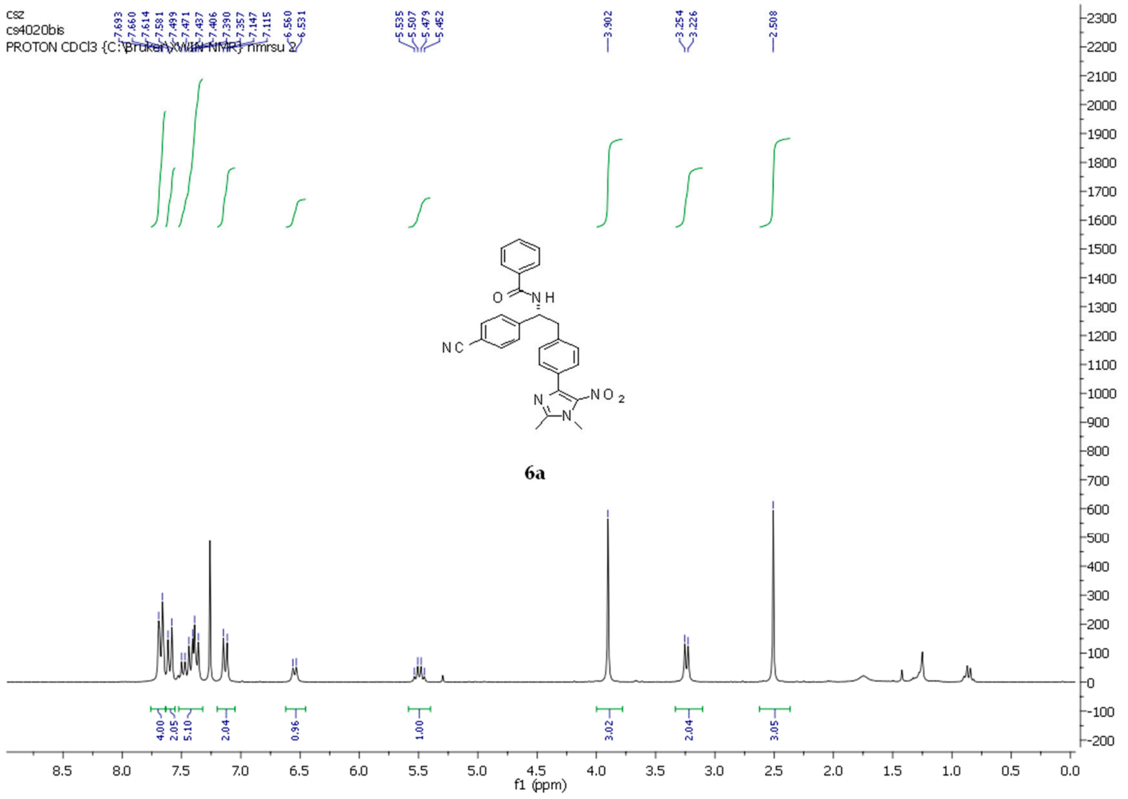

Figure S5. ^{1}H-NMR spectra of **6a**.

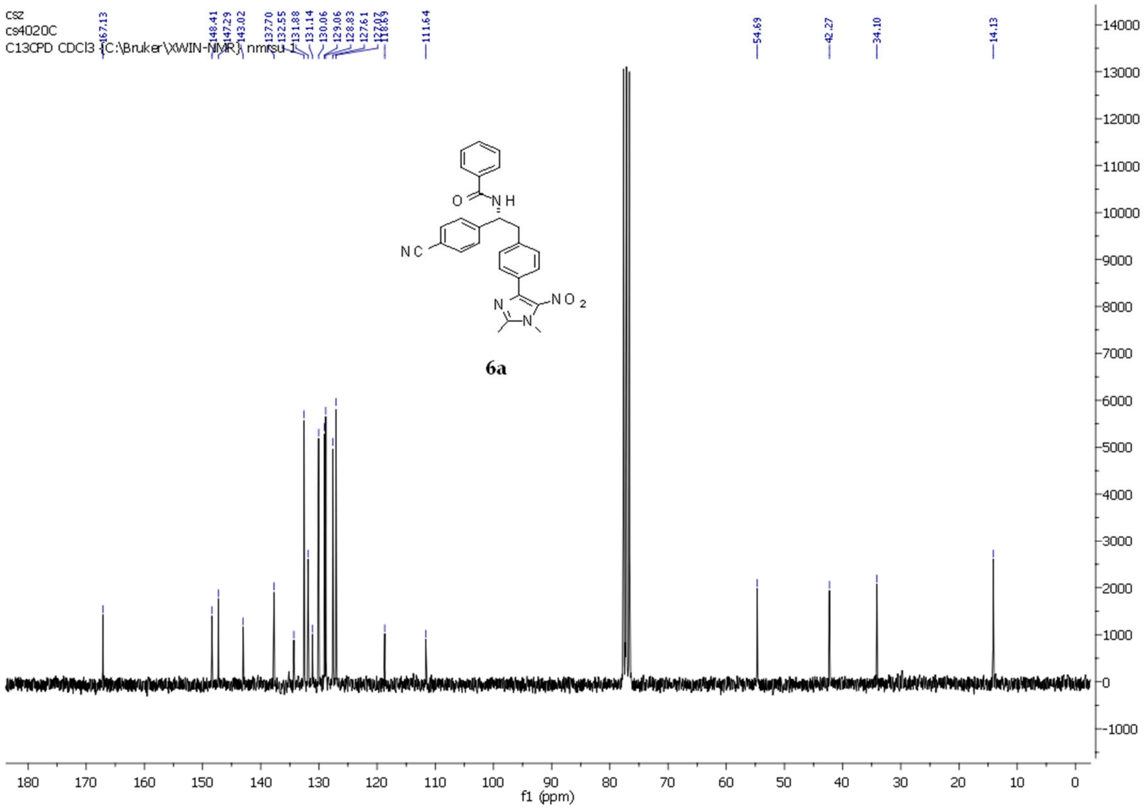

Figure S6. ^{13}C-NMR spectra of **6a**.

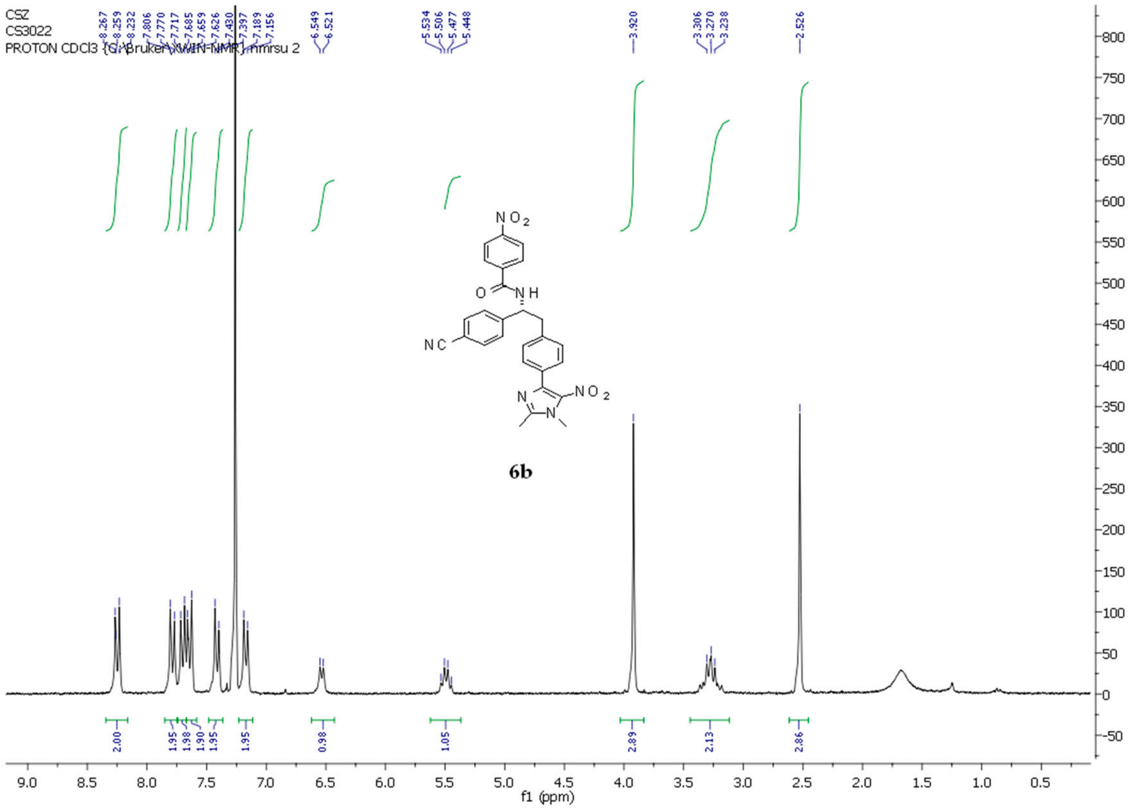

Figure S7. ^{1}H-NMR spectra of **6b**.

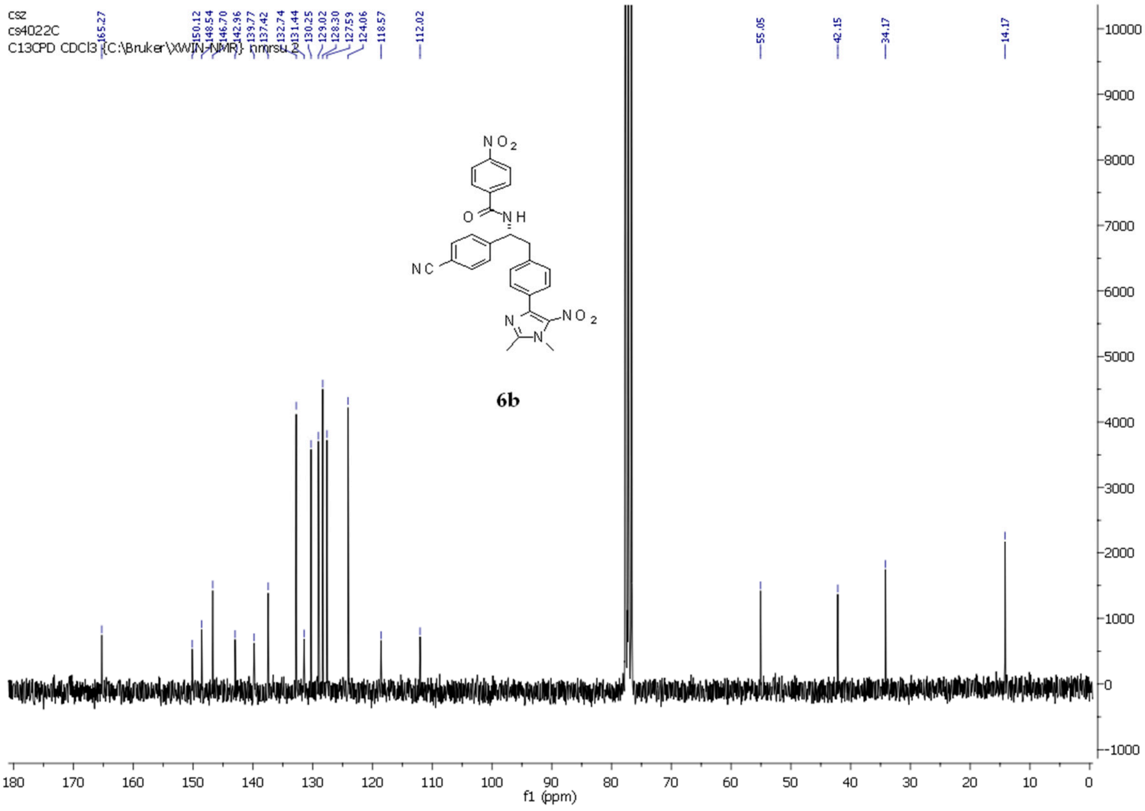

Figure S8. ^{13}C-NMR spectra of **6b**.

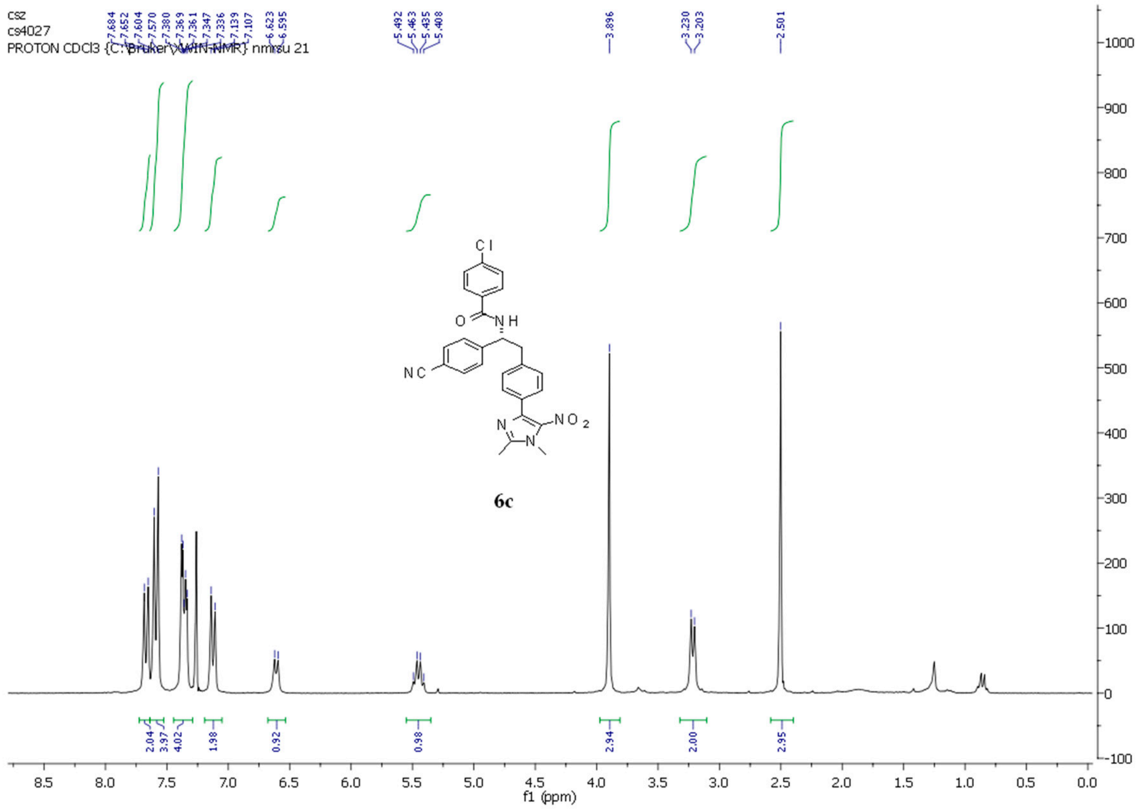

Figure S9. ^{1}H-NMR spectra of **6c**.

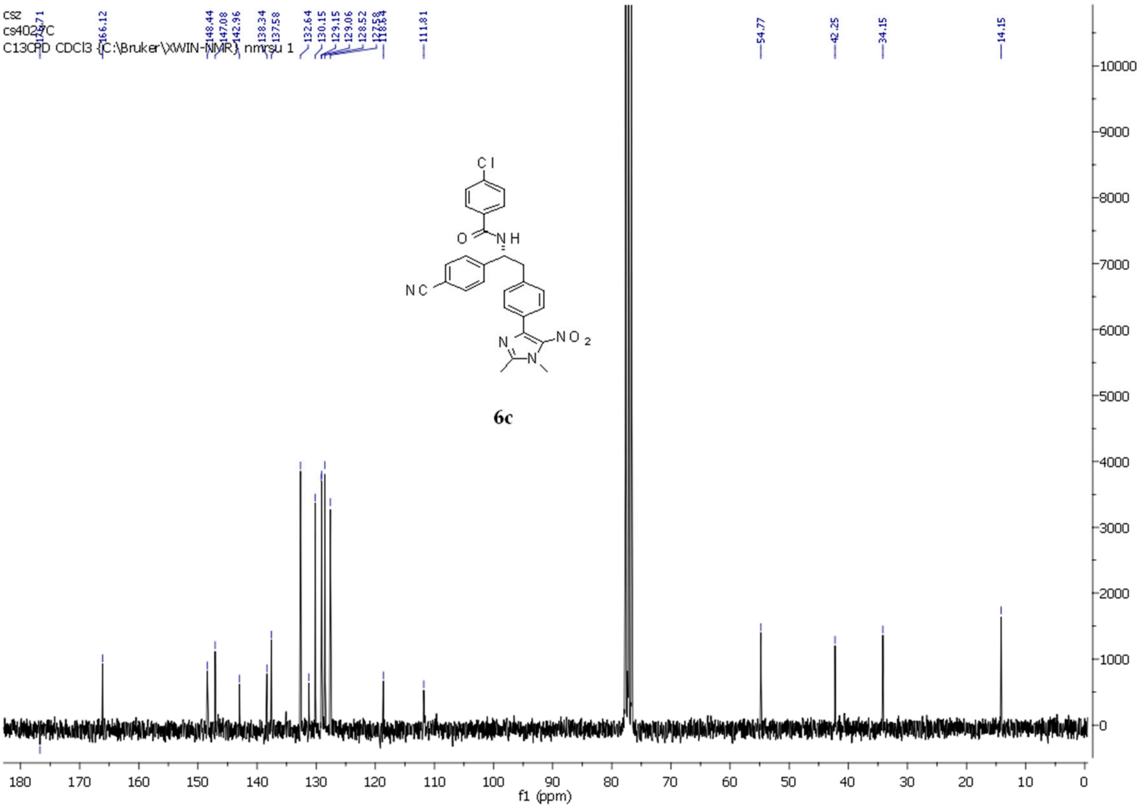

Figure S10. ^{13}C-NMR spectra of **6c**.

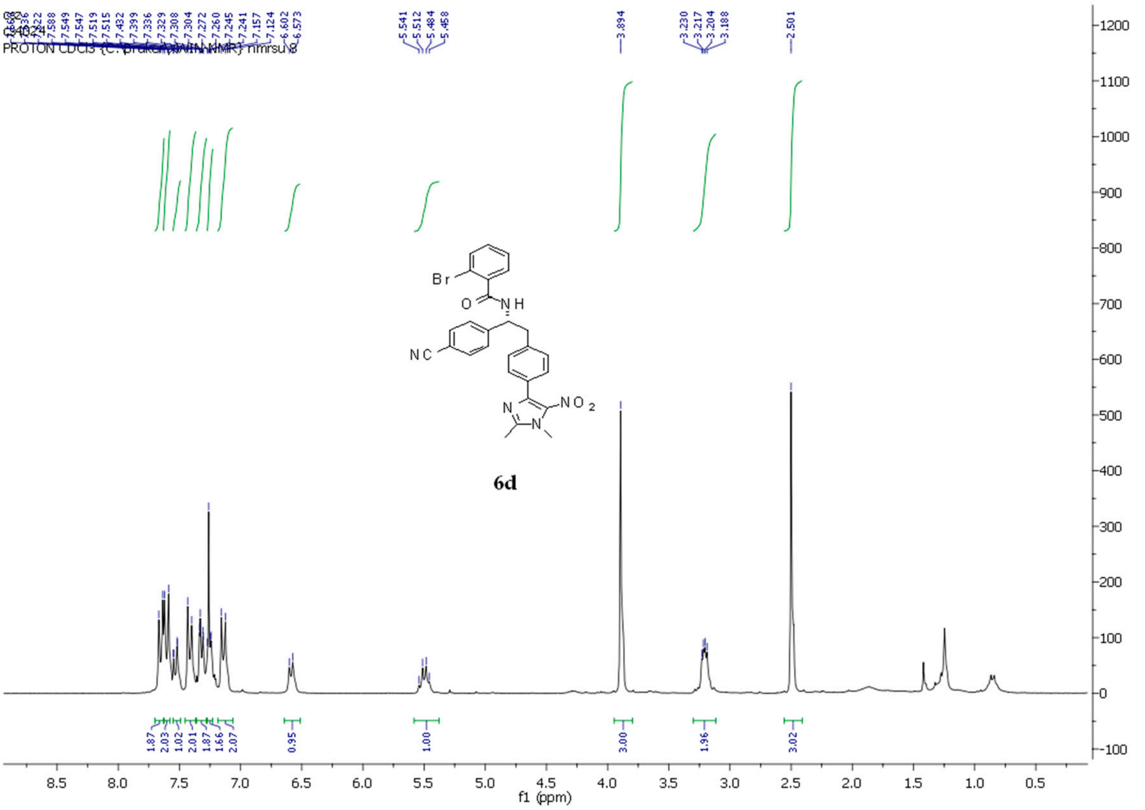

Figure S11. ^{1}H-NMR spectra of **6d**.

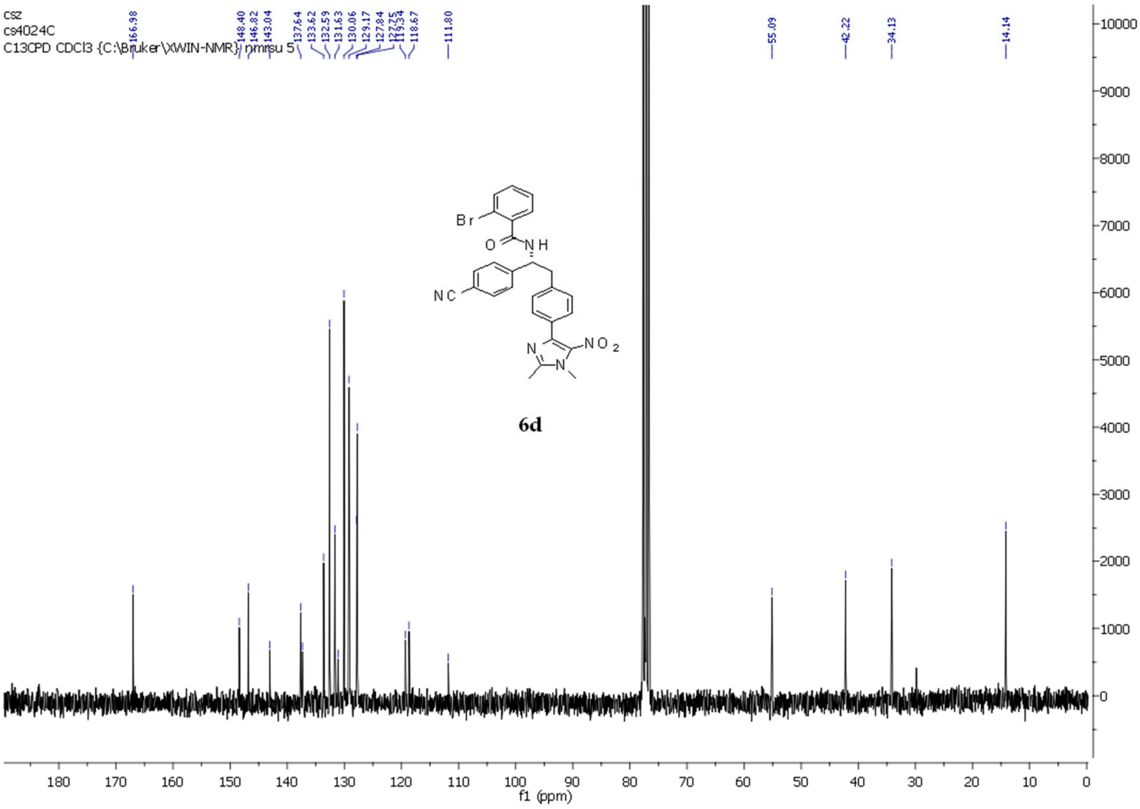

Figure S12. ^{13}C-NMR spectra of **6d**.

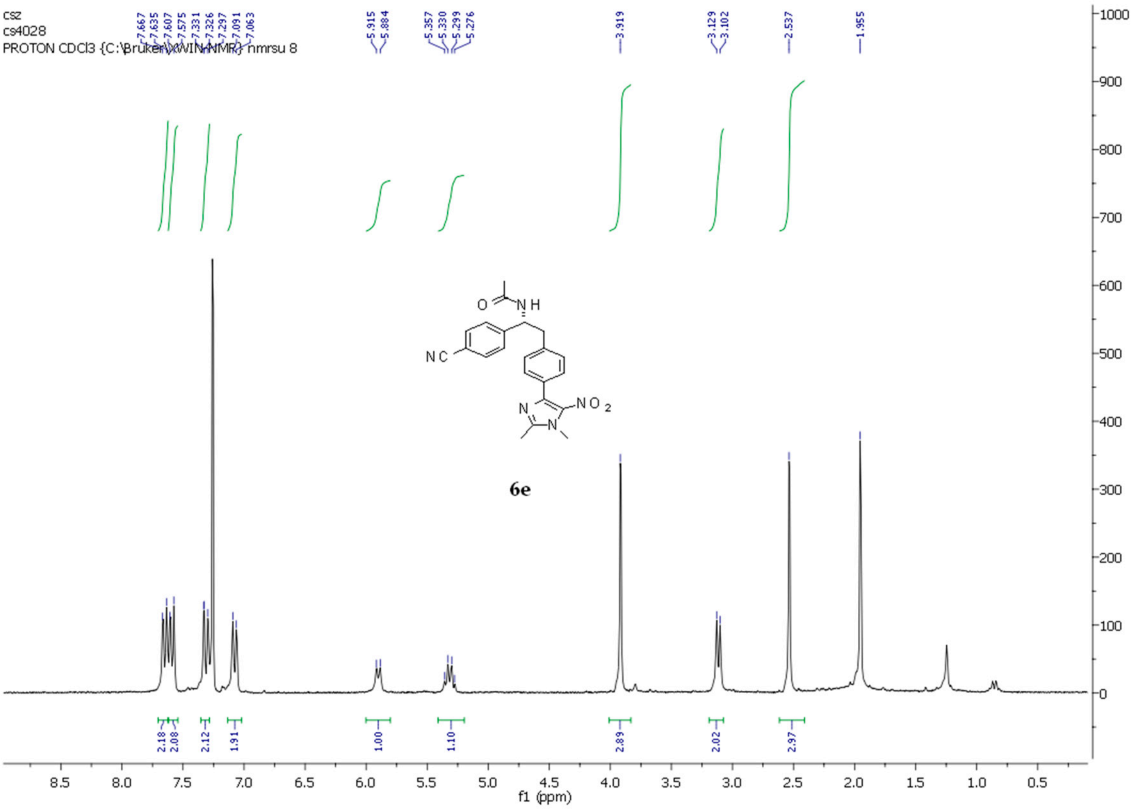

Figure S13. ^{1}H-NMR spectra of **6e**.

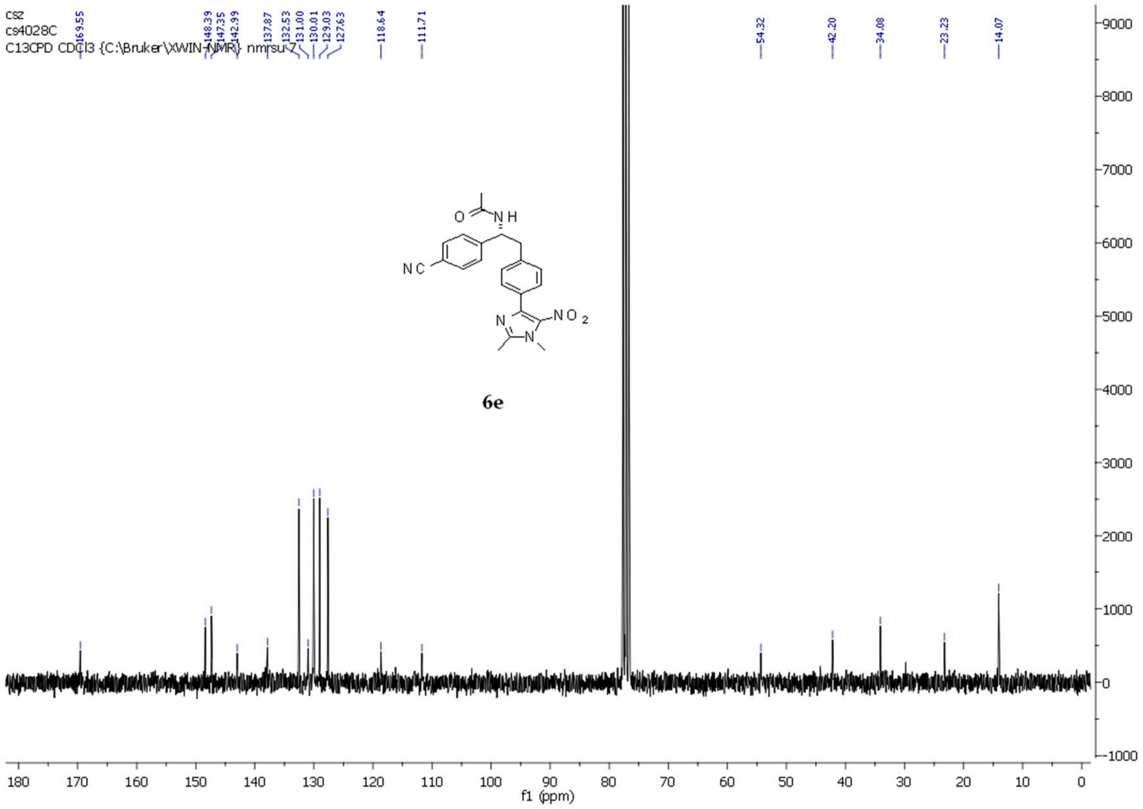

Figure S14. ^{13}C-NMR spectra of **6e**.

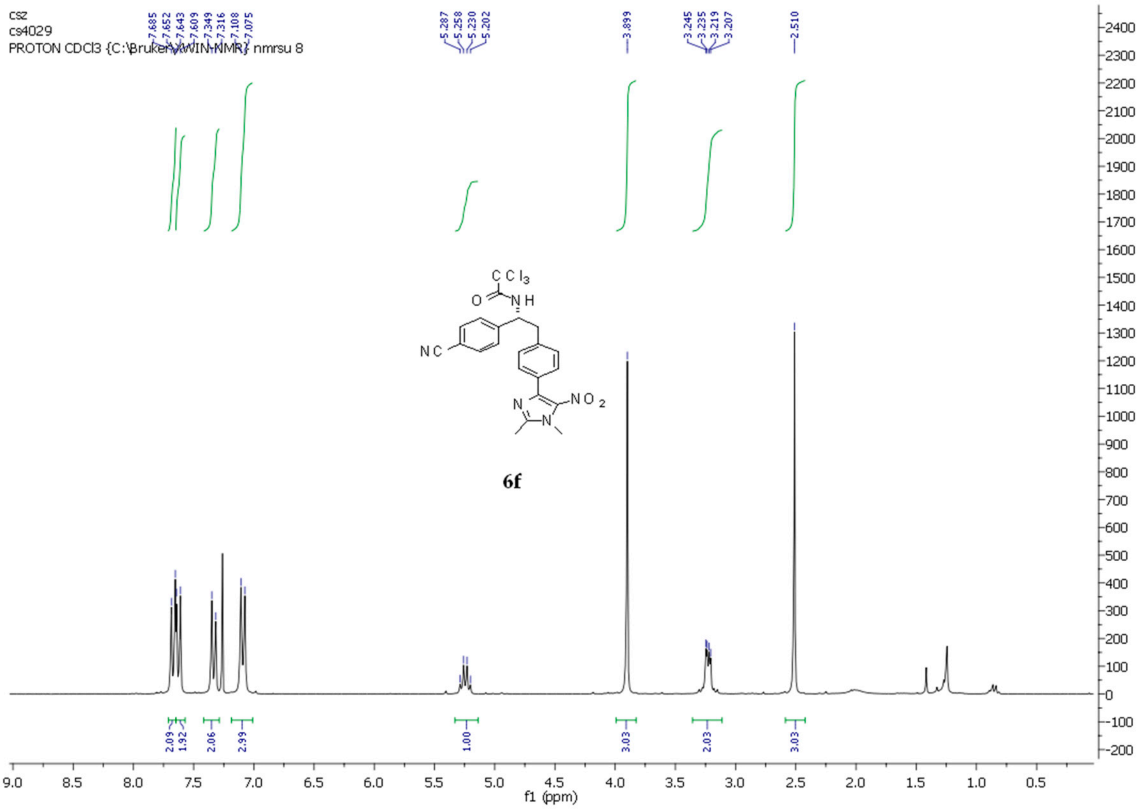

Figure S15. ^{1}H-NMR spectra of **6f**.

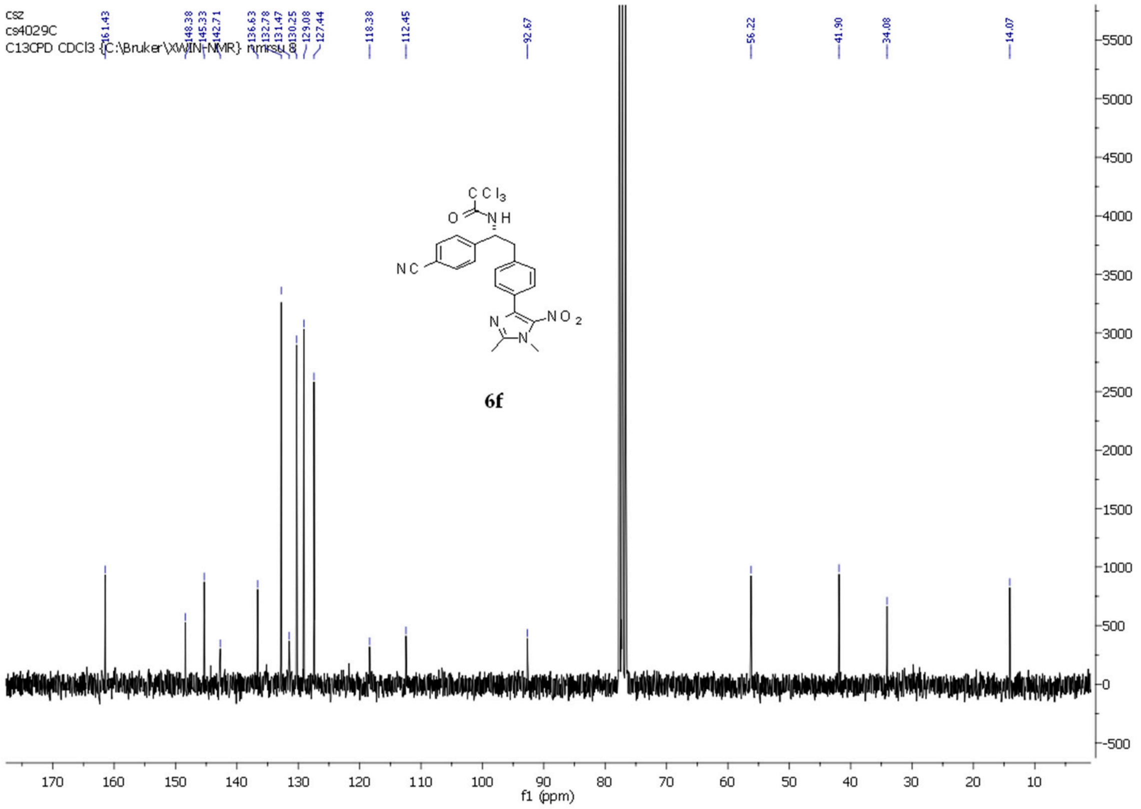

Figure S16. ^{13}C-NMR spectra of **6f**.

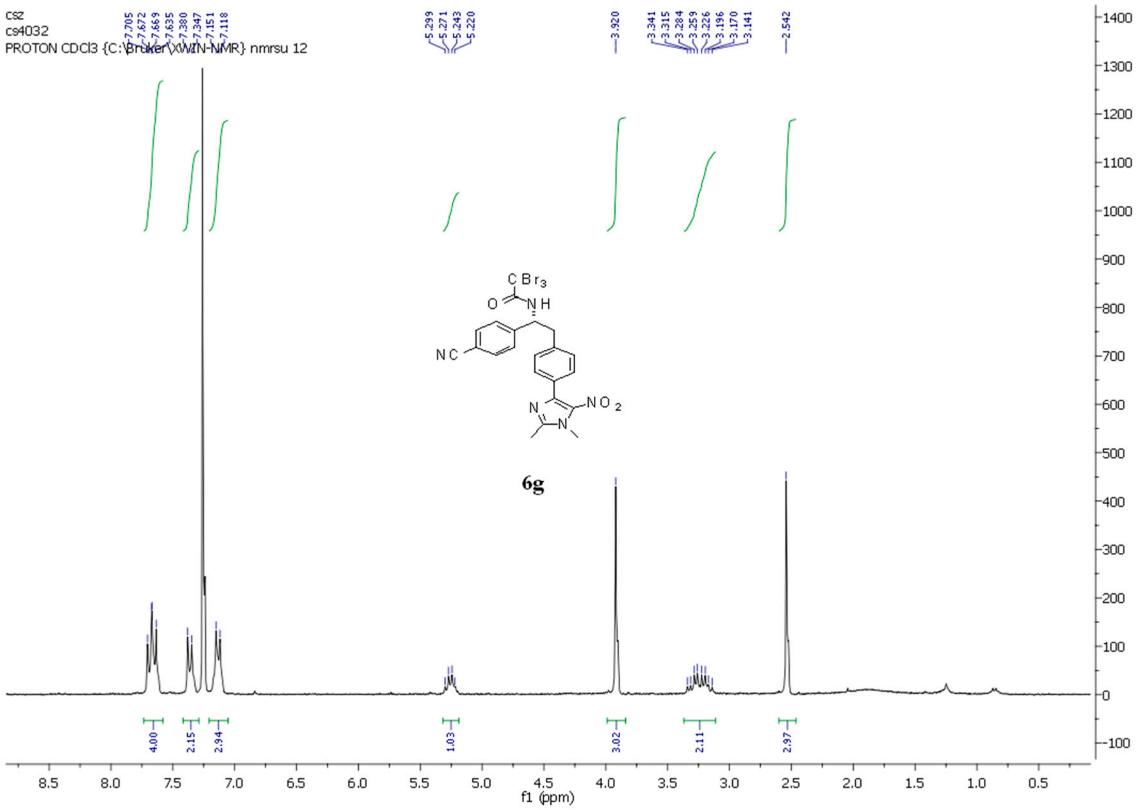

Figure S17. ^{1}H-NMR spectra of **6g**.

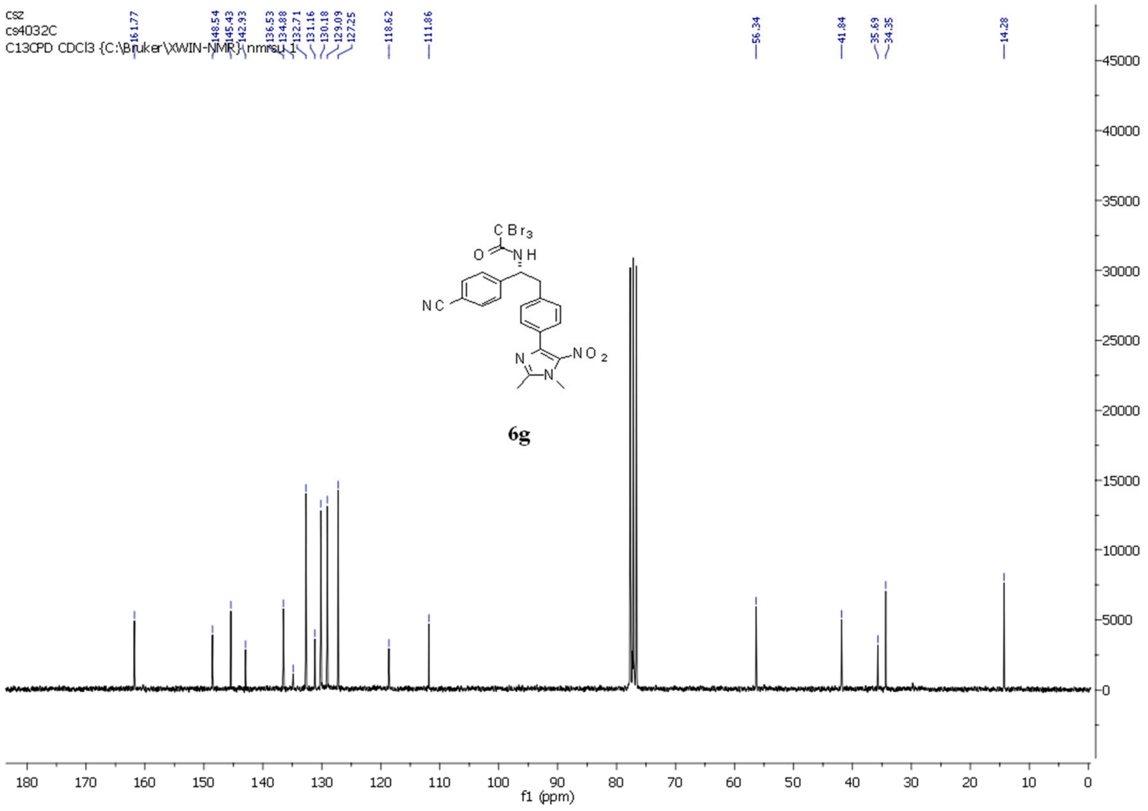

Figure S18. ^{13}C-NMR spectra of **6g**.

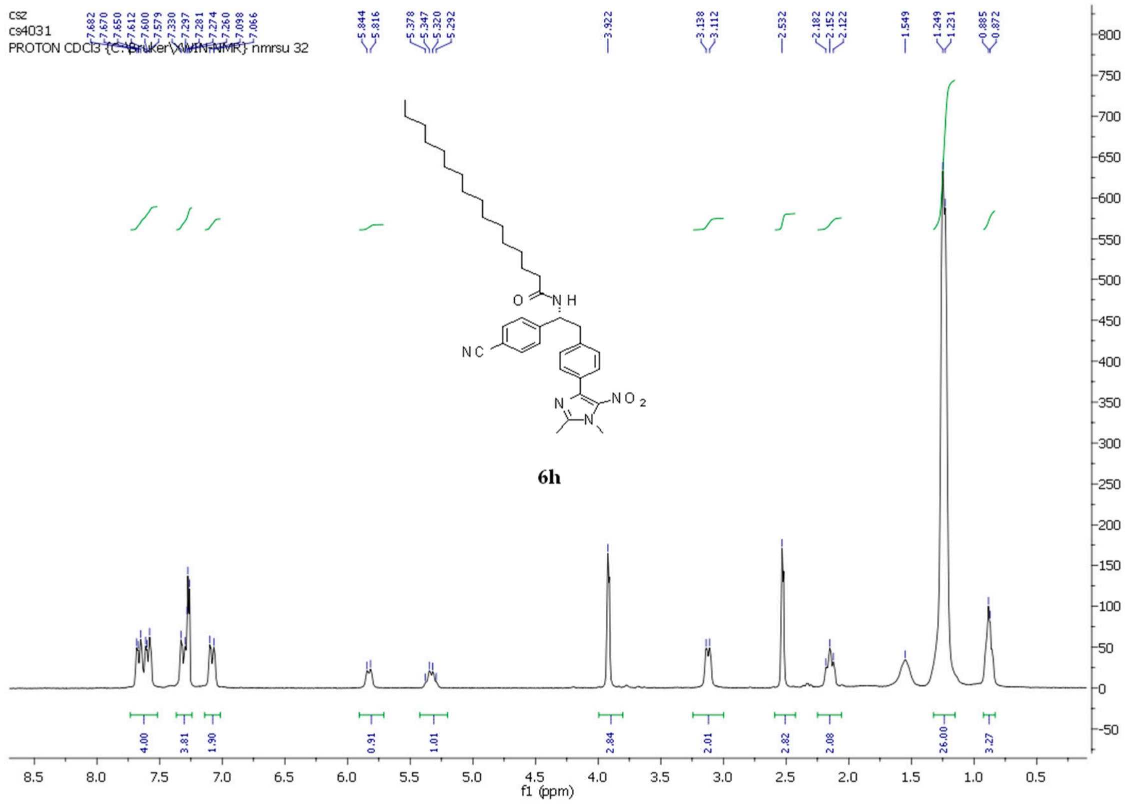

Figure S19. ^{1}H-NMR spectra of **6h**.

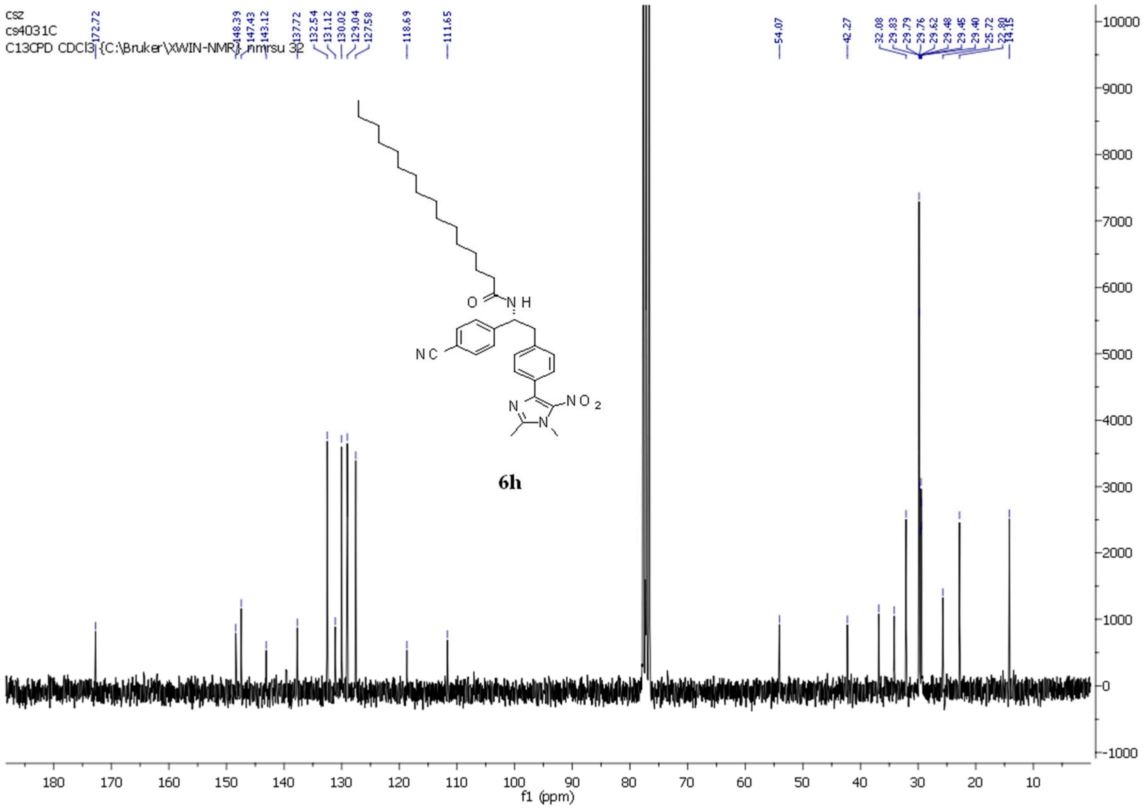

Figure S20. ^{13}C-NMR spectra of **6h**.

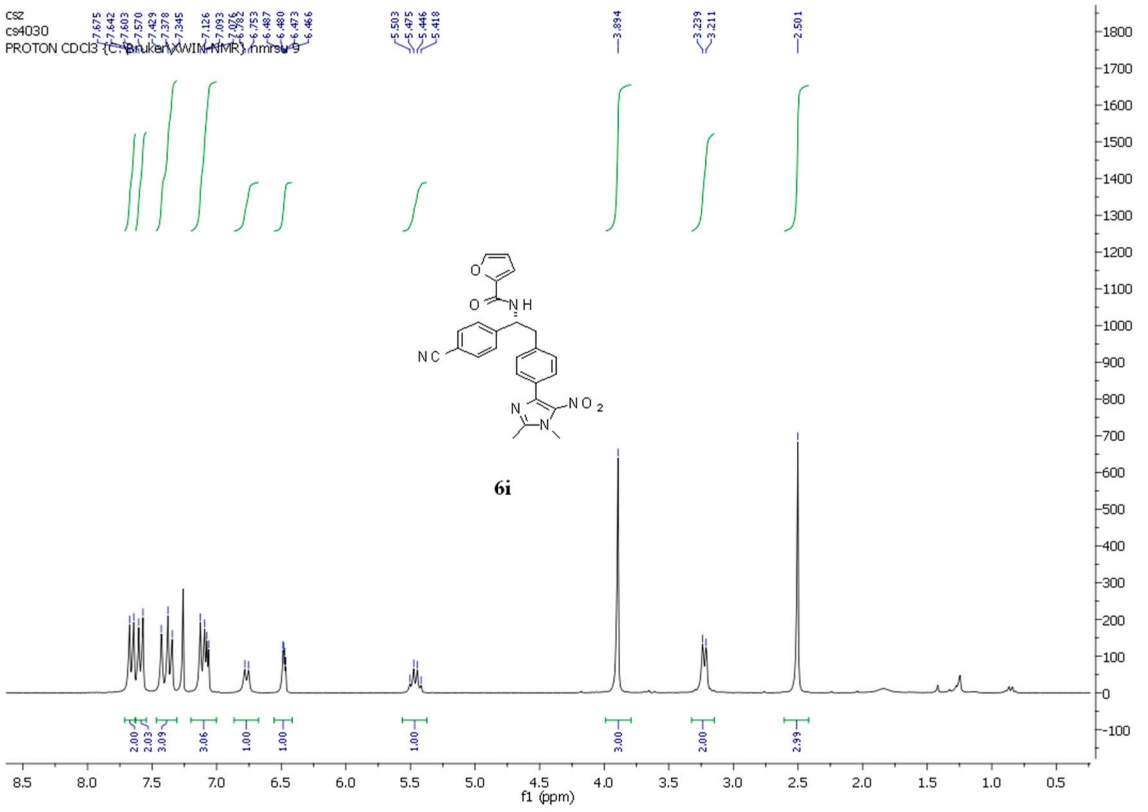

Figure S21. ^{1}H-NMR spectra of **6i**.

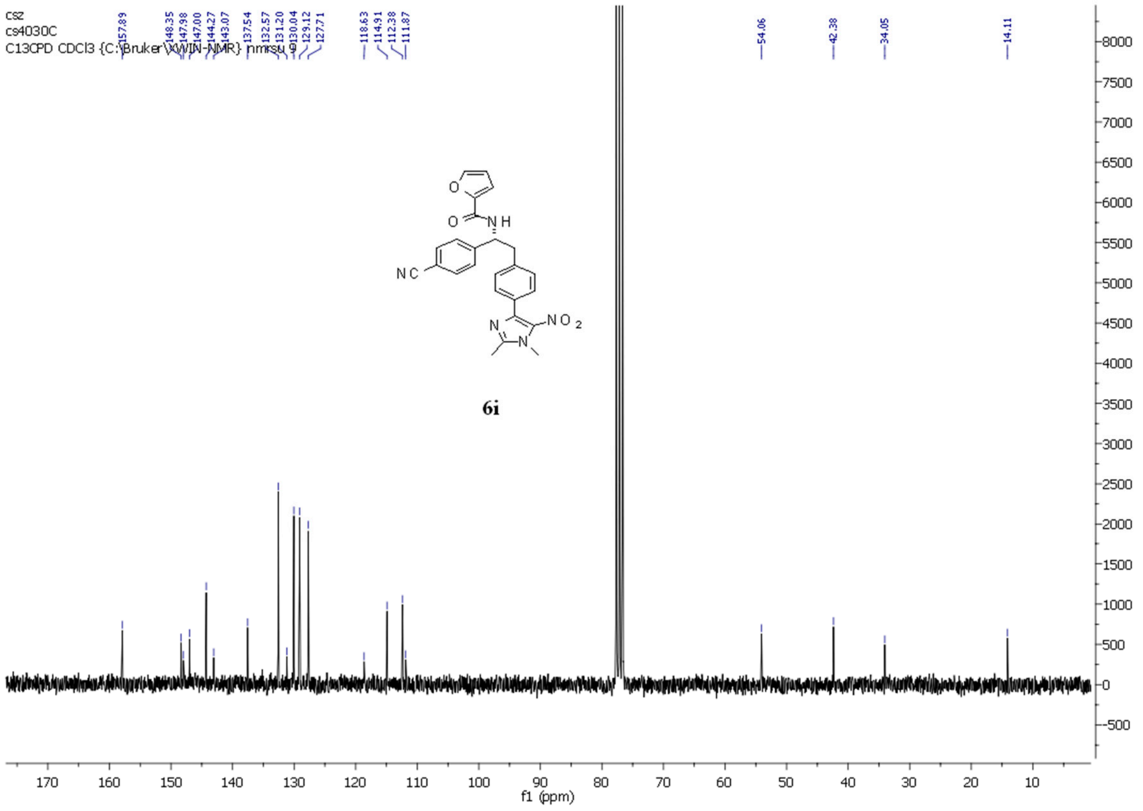

Figure S22. ^{13}C-NMR spectra of **6i**.

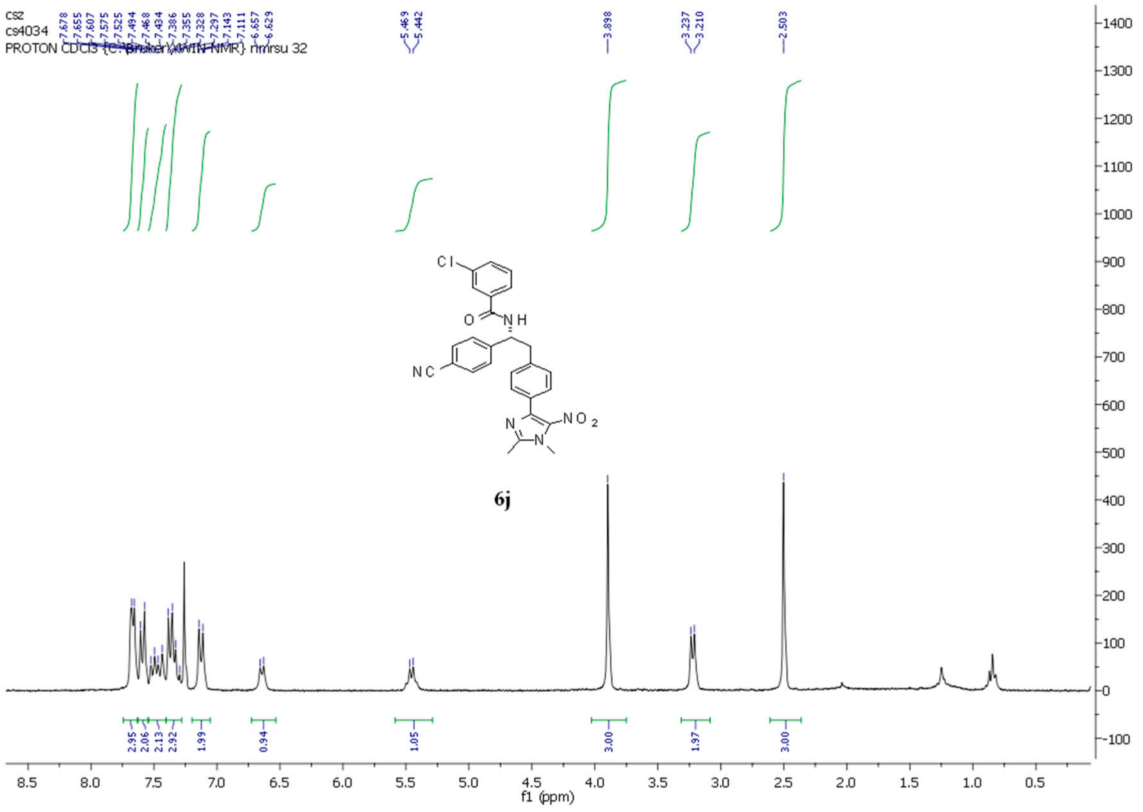

Figure S23. ^{1}H-NMR spectra of **6j**.

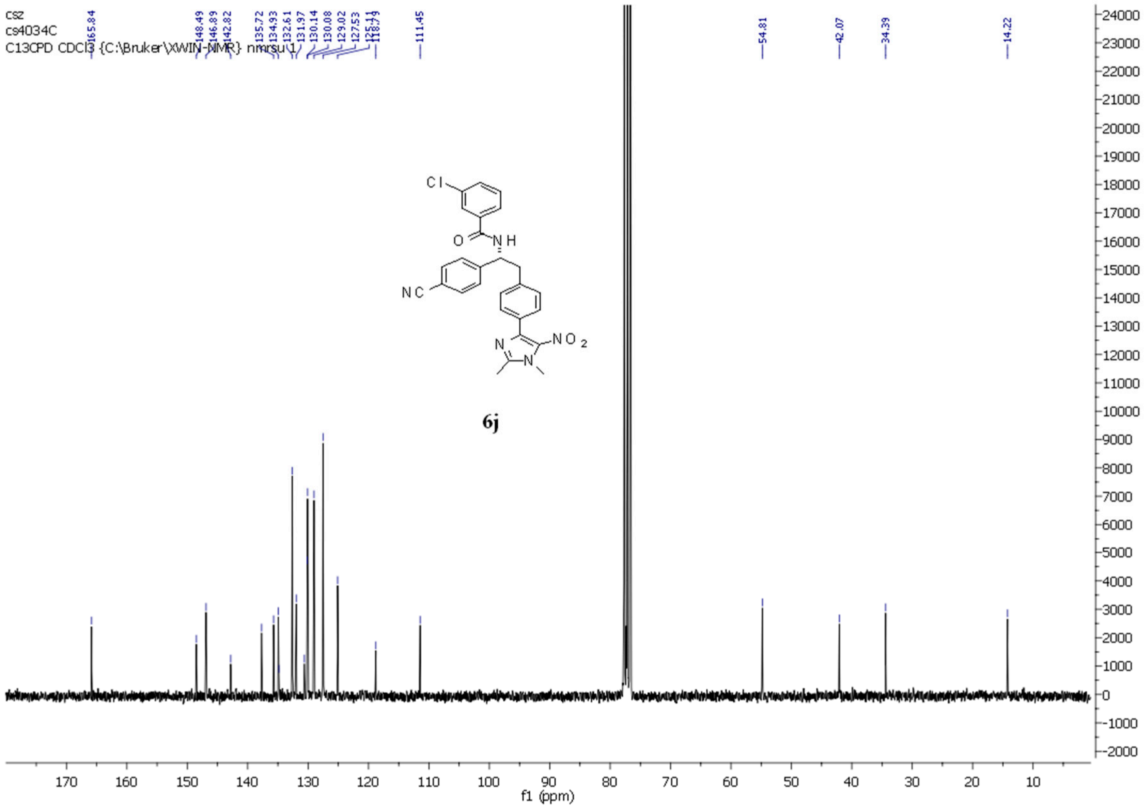

Figure S24. ^{13}C-NMR spectra of **6j**.

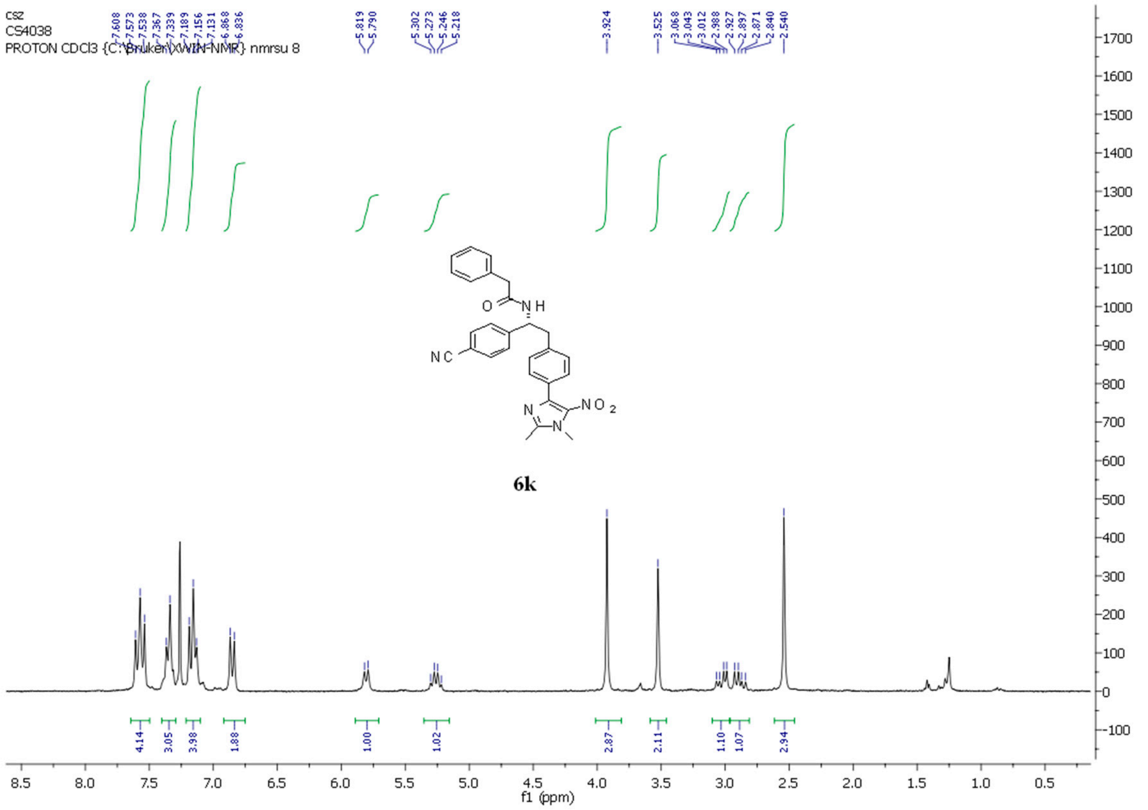

Figure S25. ^{1}H-NMR spectra of **6k**.

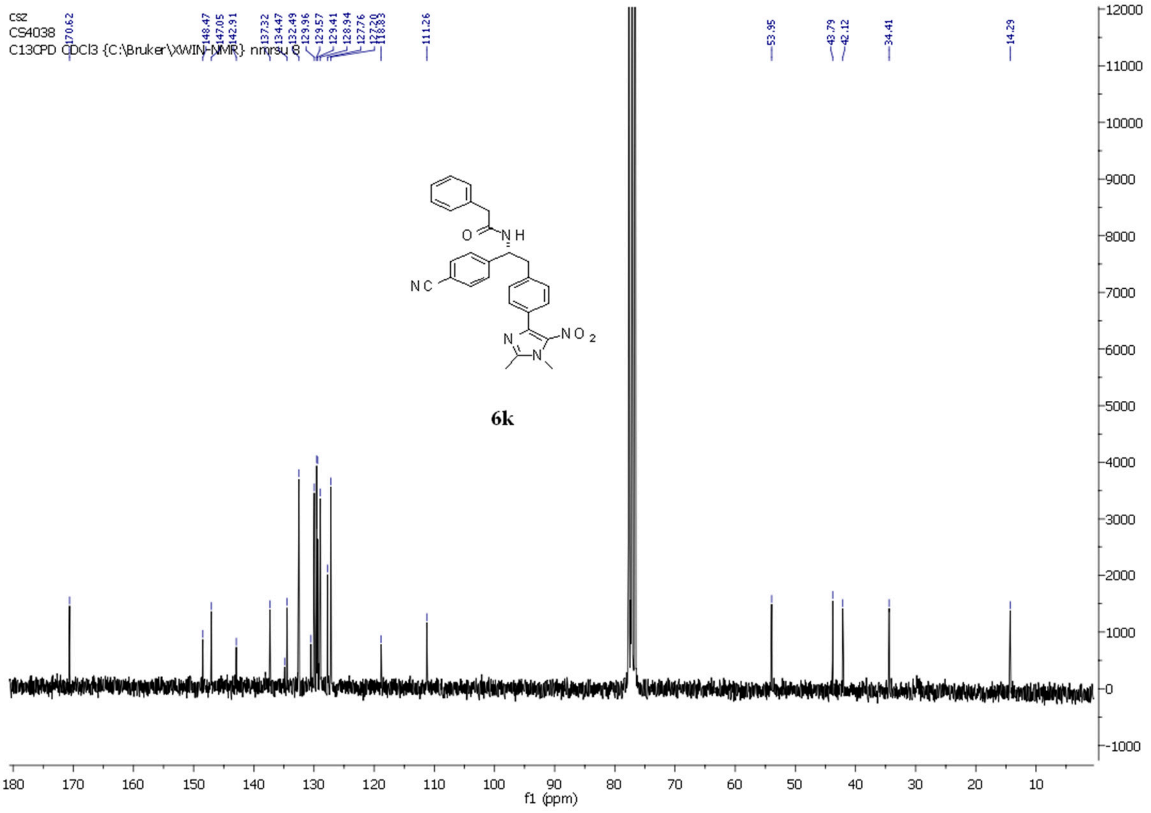

Figure S26. ^{13}C-NMR spectra of **6k**.

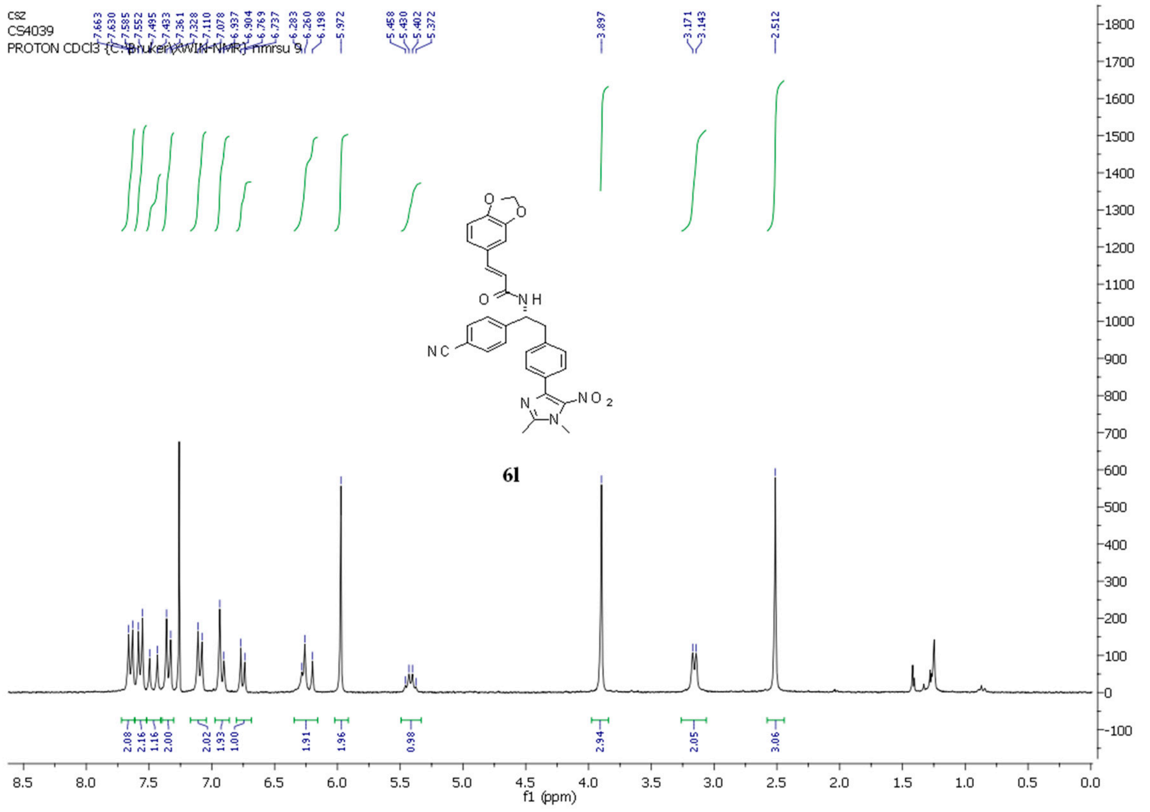

Figure S27. ^{1}H-NMR spectra of **6l**.

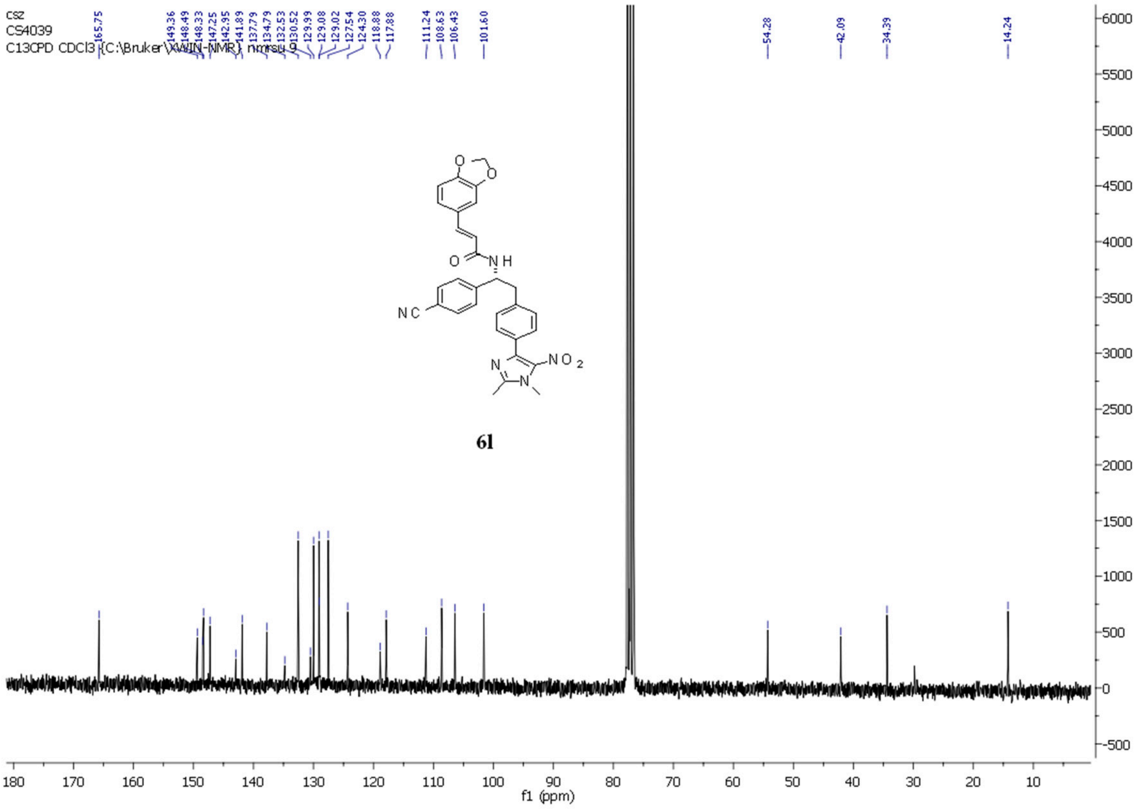

Figure S28. ^{13}C-NMR spectra of **6l**.

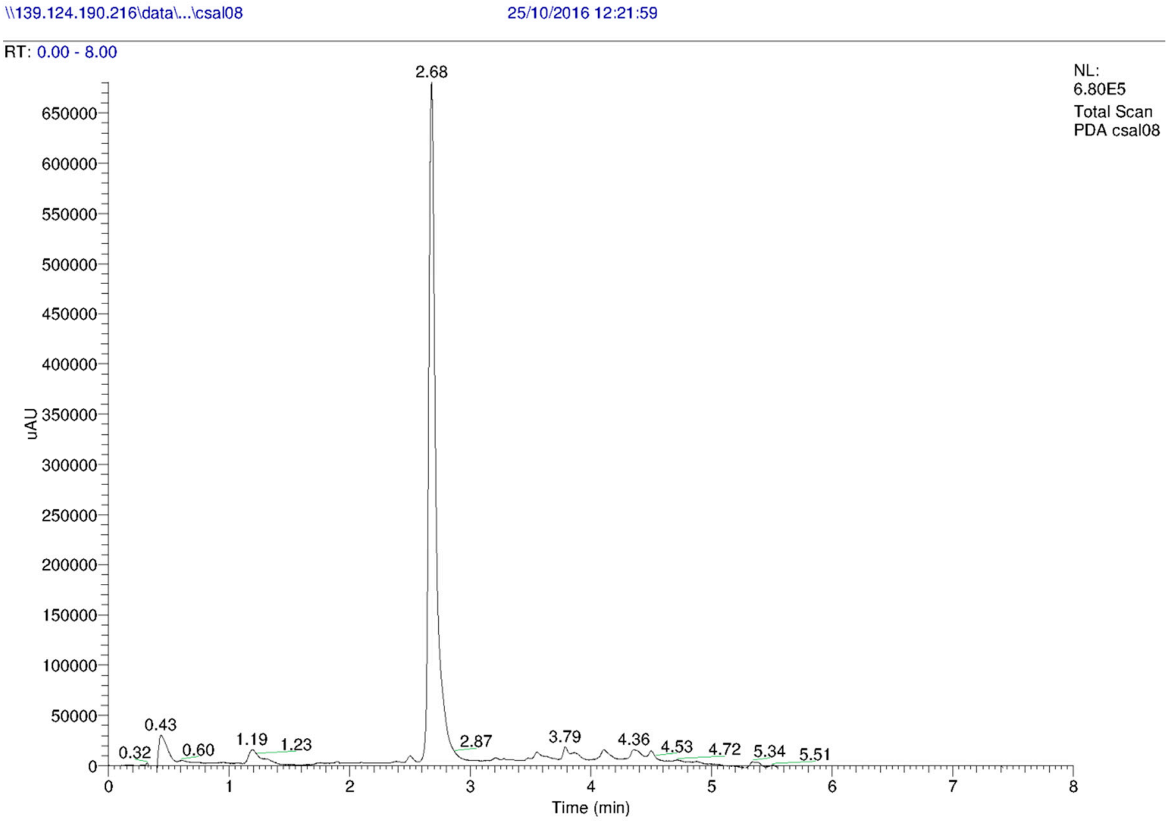

Figure S29. HPLC spectra of **3**.

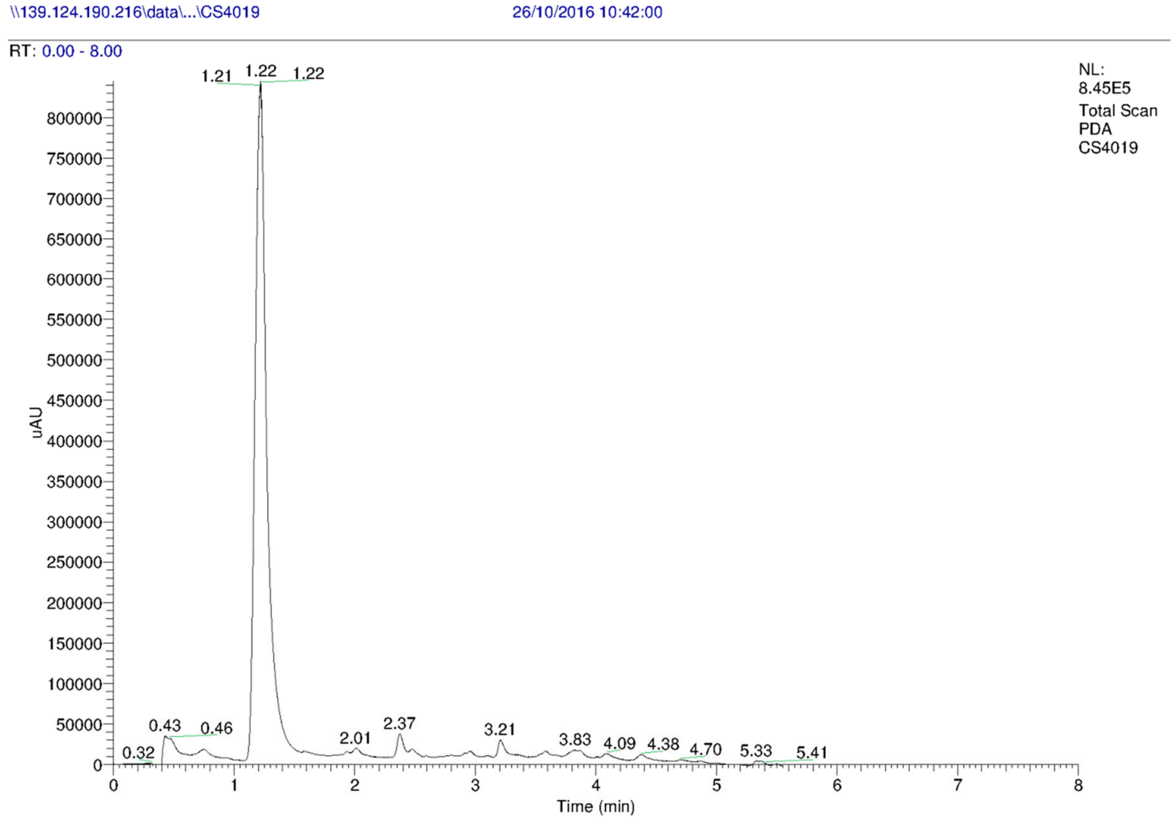

Figure S30. HPLC spectra of **4**.

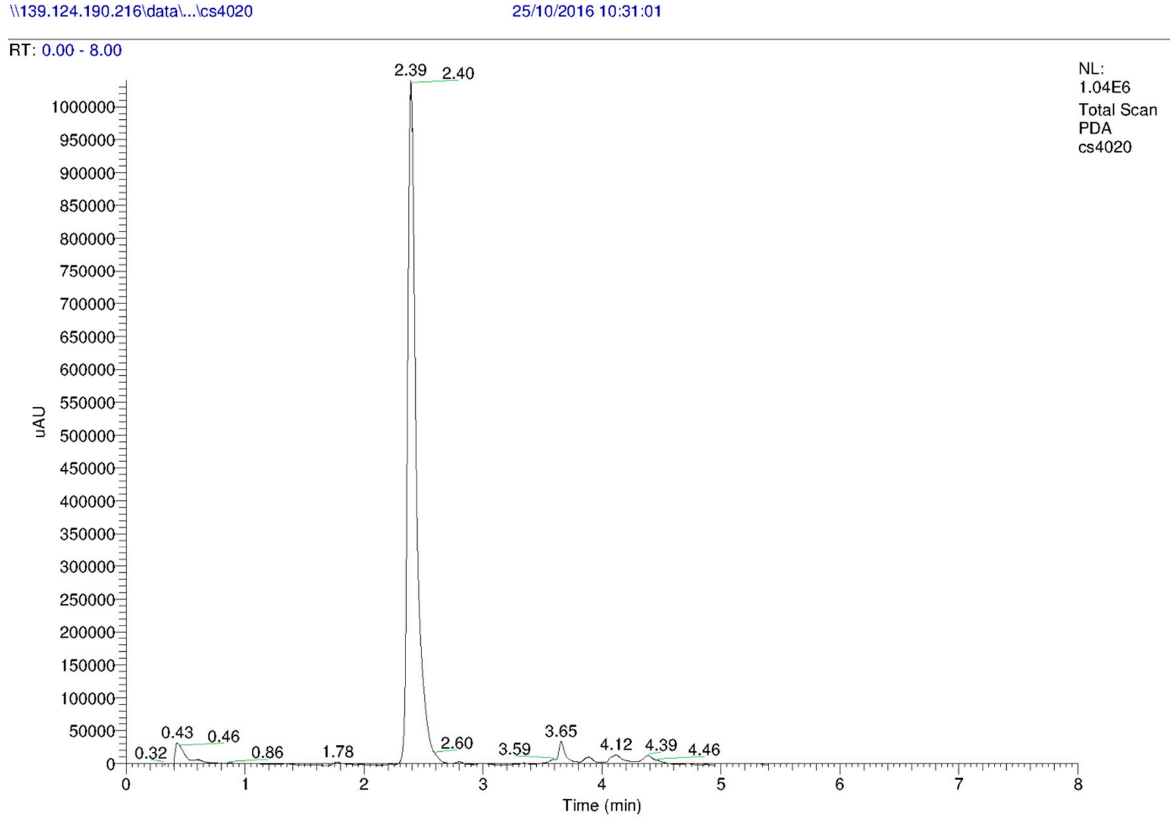

Figure S31. HPLC spectra of **6a**.

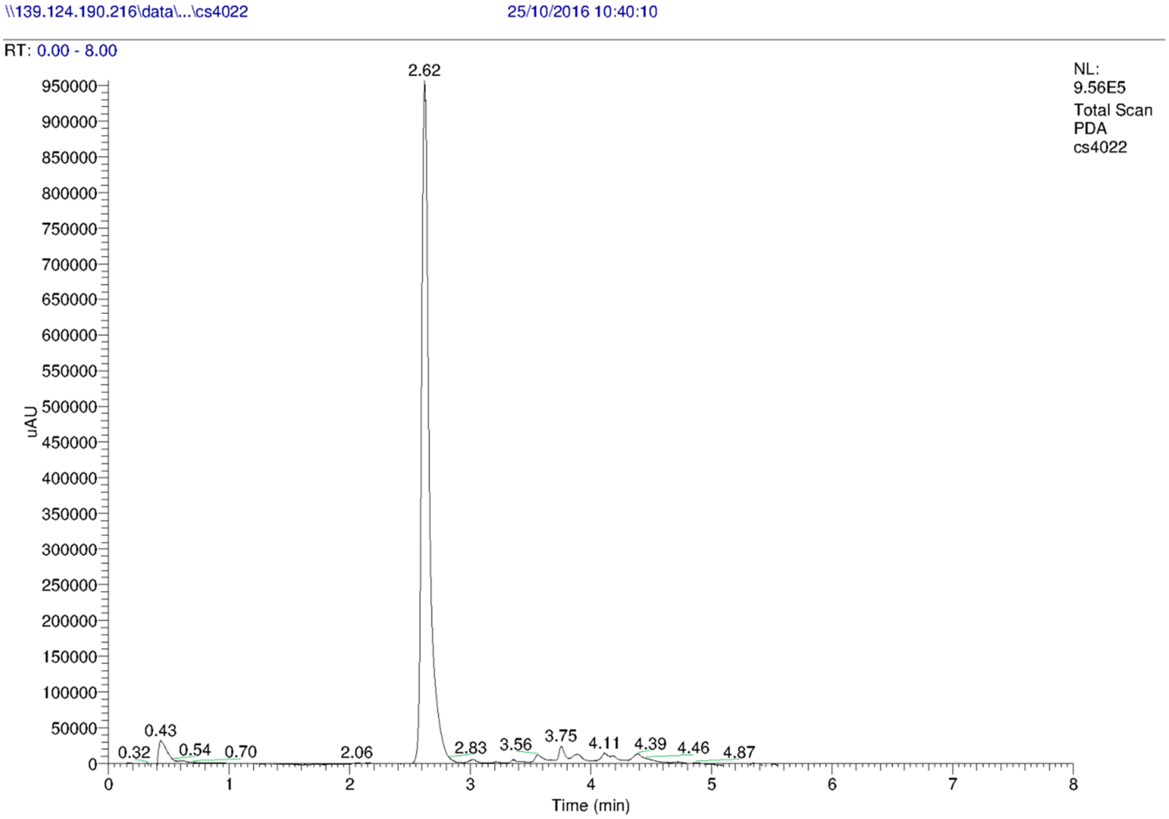

Figure S32. HPLC spectra of **6b**.

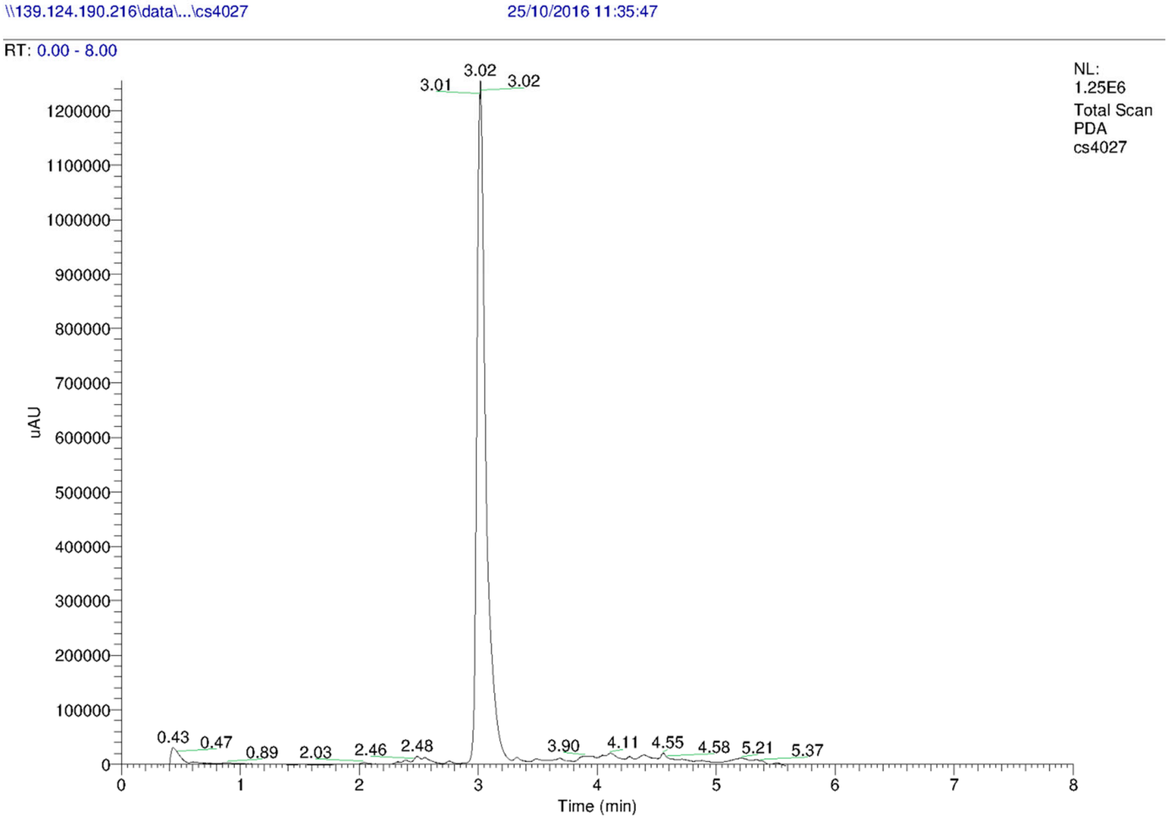

Figure S33. HPLC spectra of **6c**.

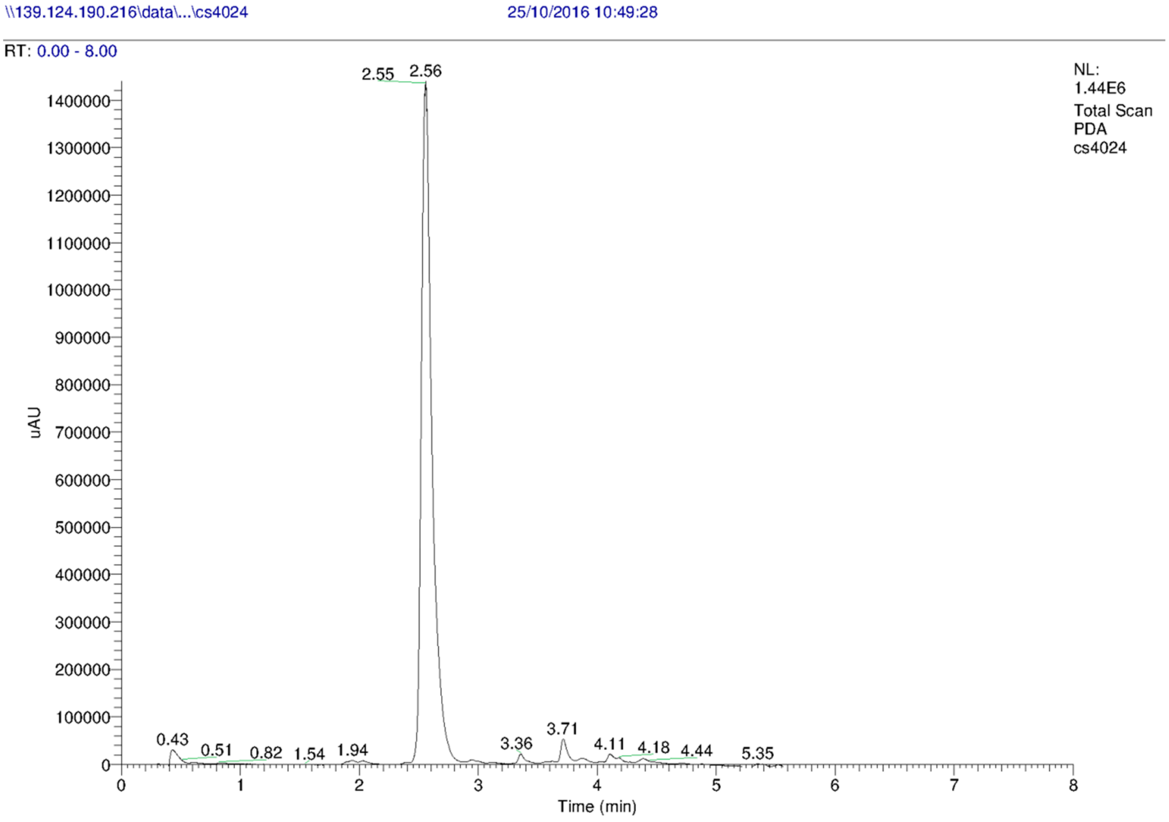

Figure S34. HPLC spectra of **6d**.

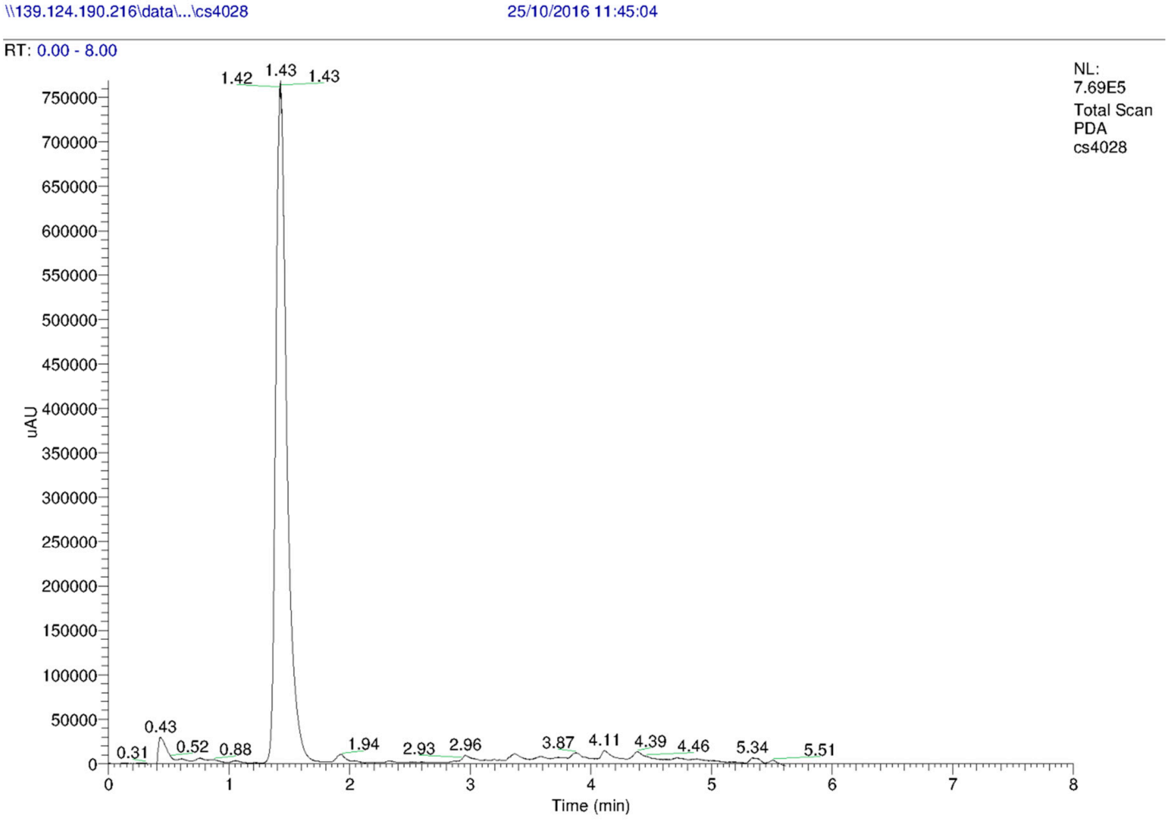

Figure S35. HPLC spectra of **6e**.

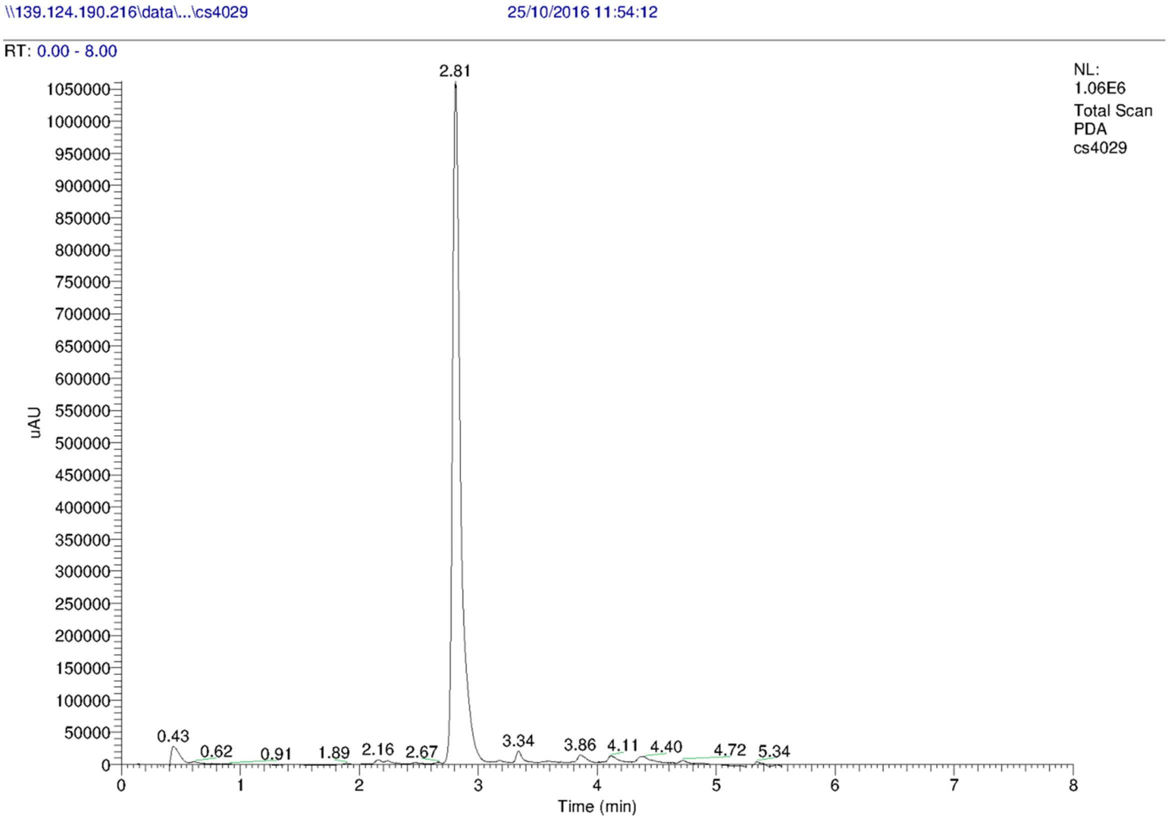

Figure S36. HPLC spectra of **6f**.

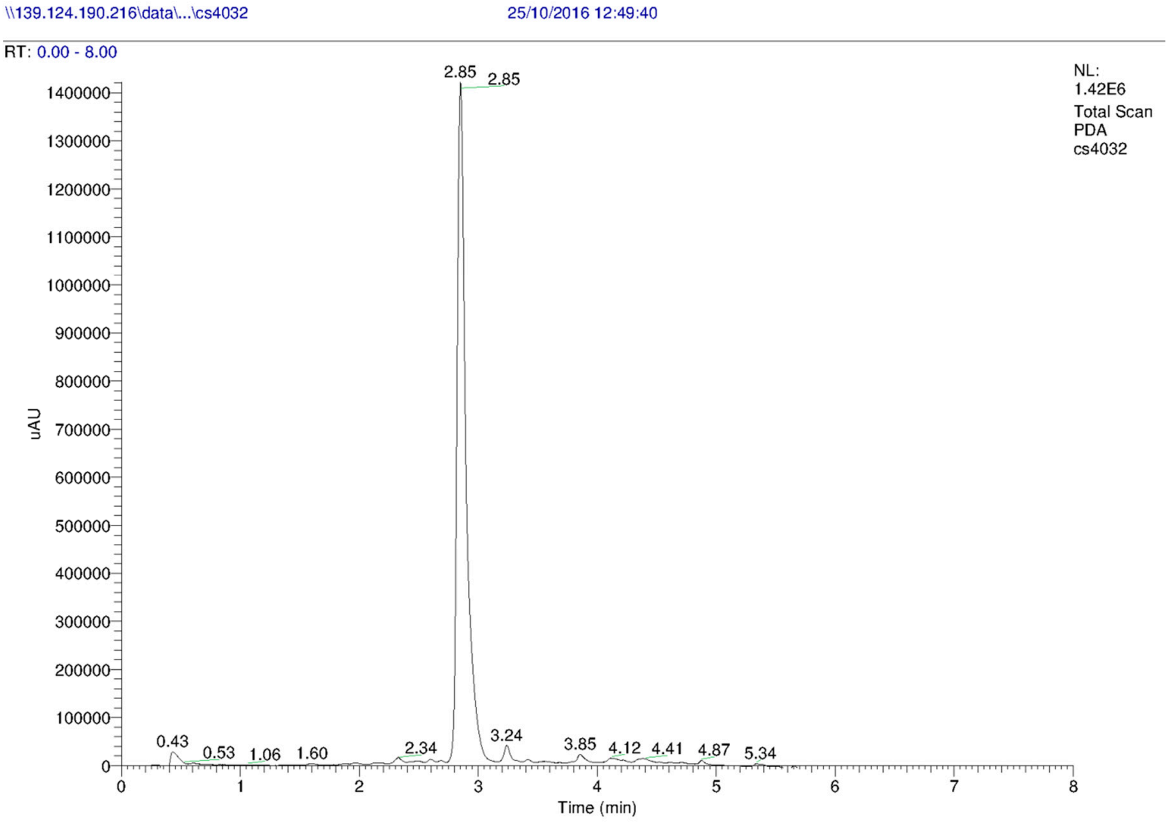

Figure S37. HPLC spectra of **6g**.

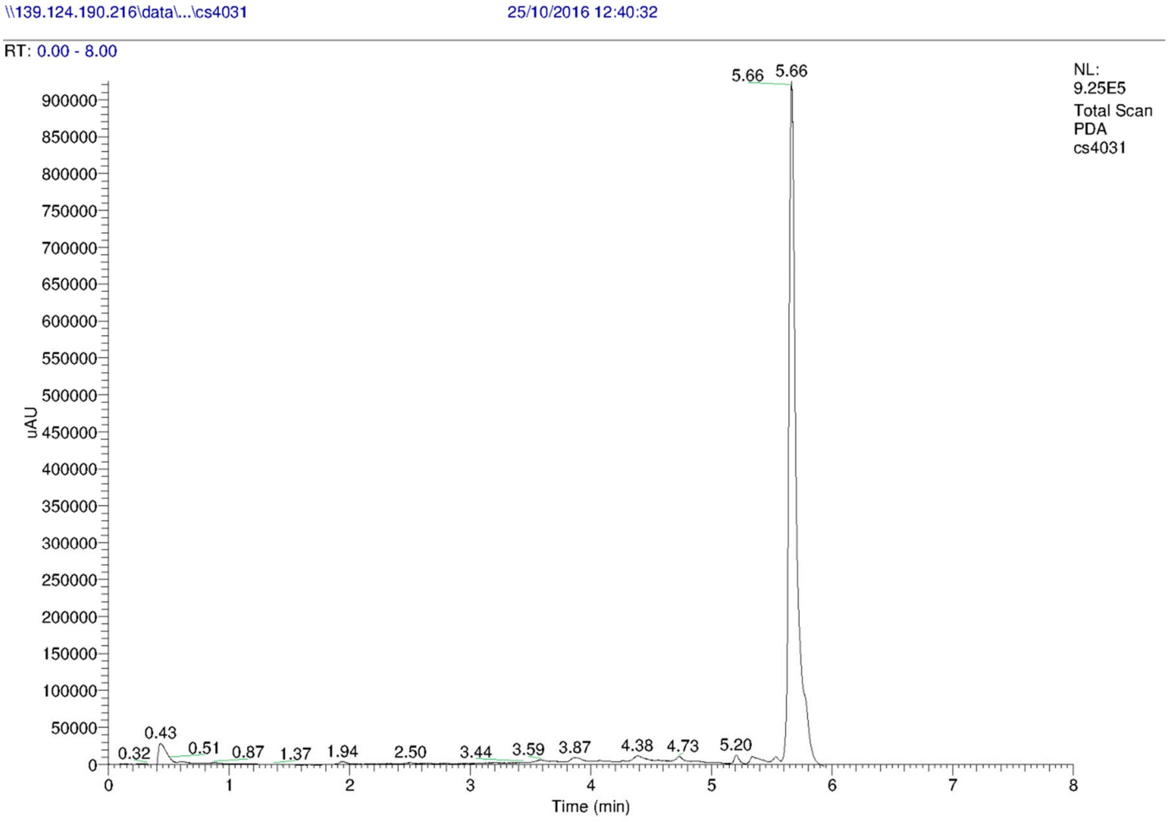

Figure S38. HPLC spectra of **6h**.

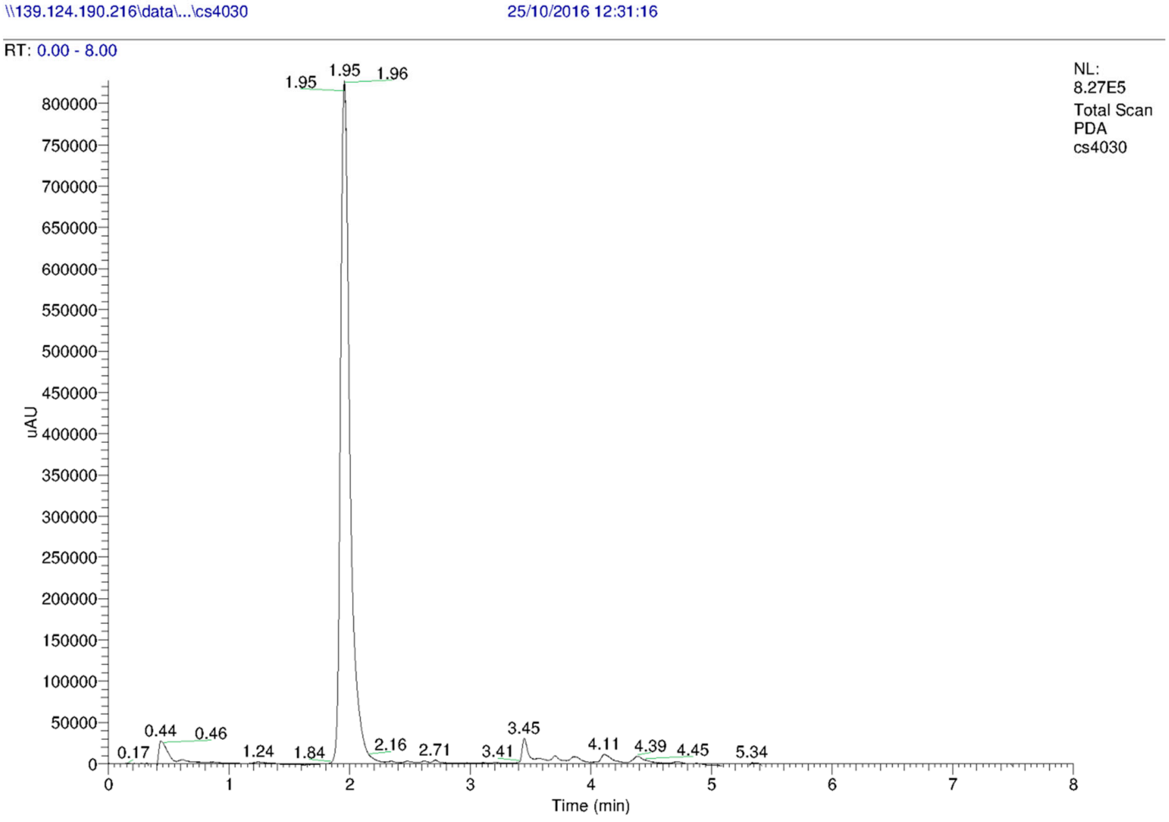

Figure S39. HPLC spectra of **6i**.

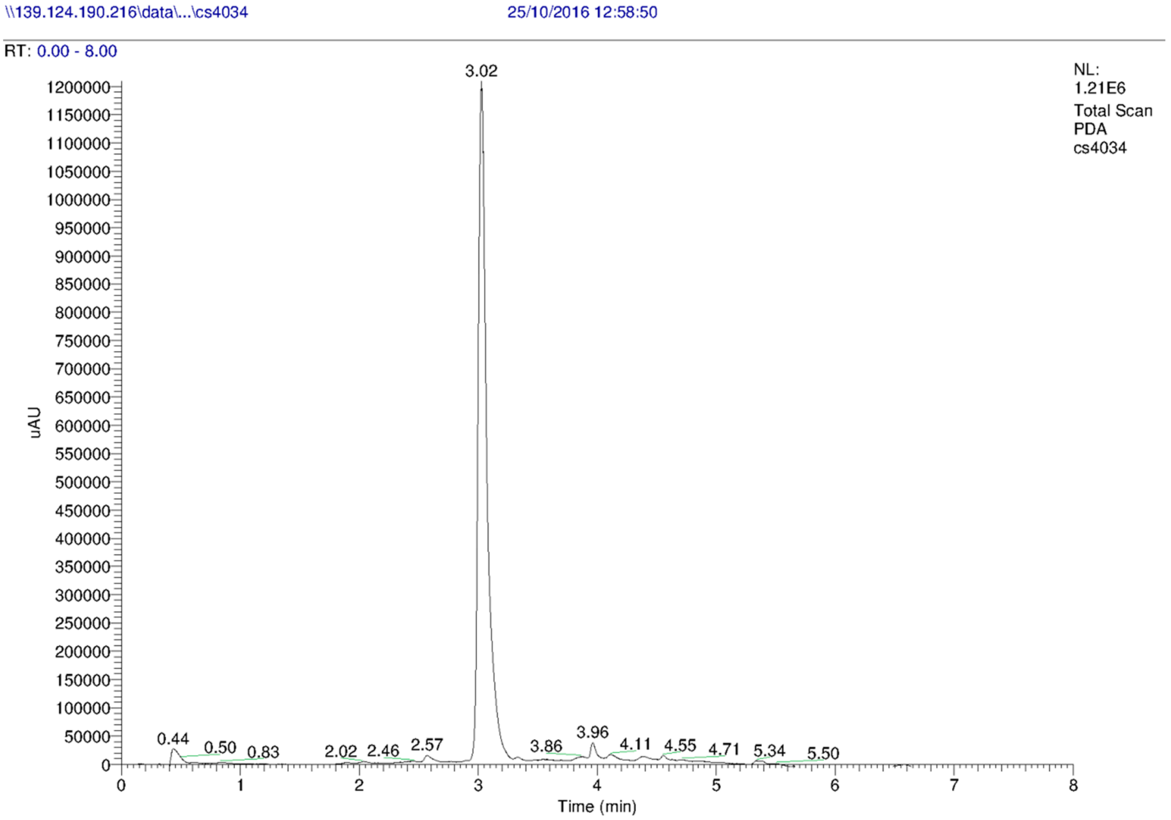

Figure S40. HPLC spectra of **6j**.

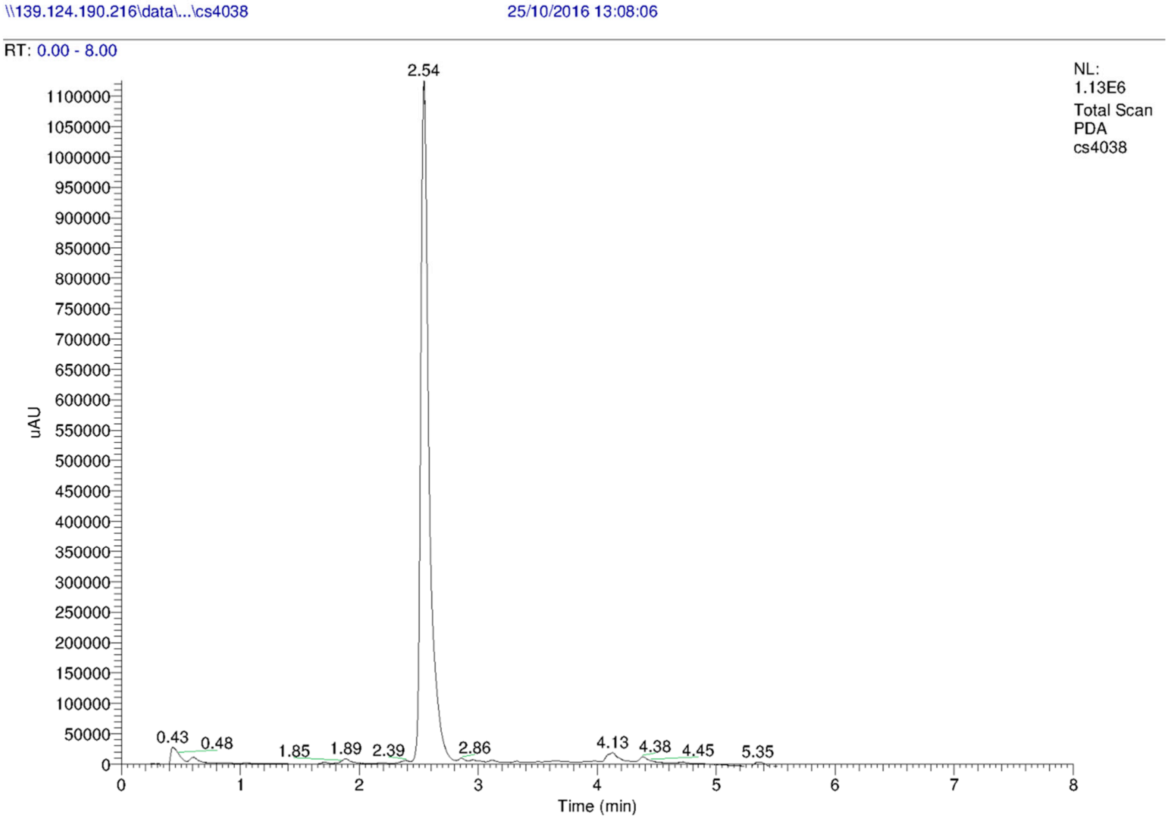

Figure S41. HPLC spectra of **6k**.

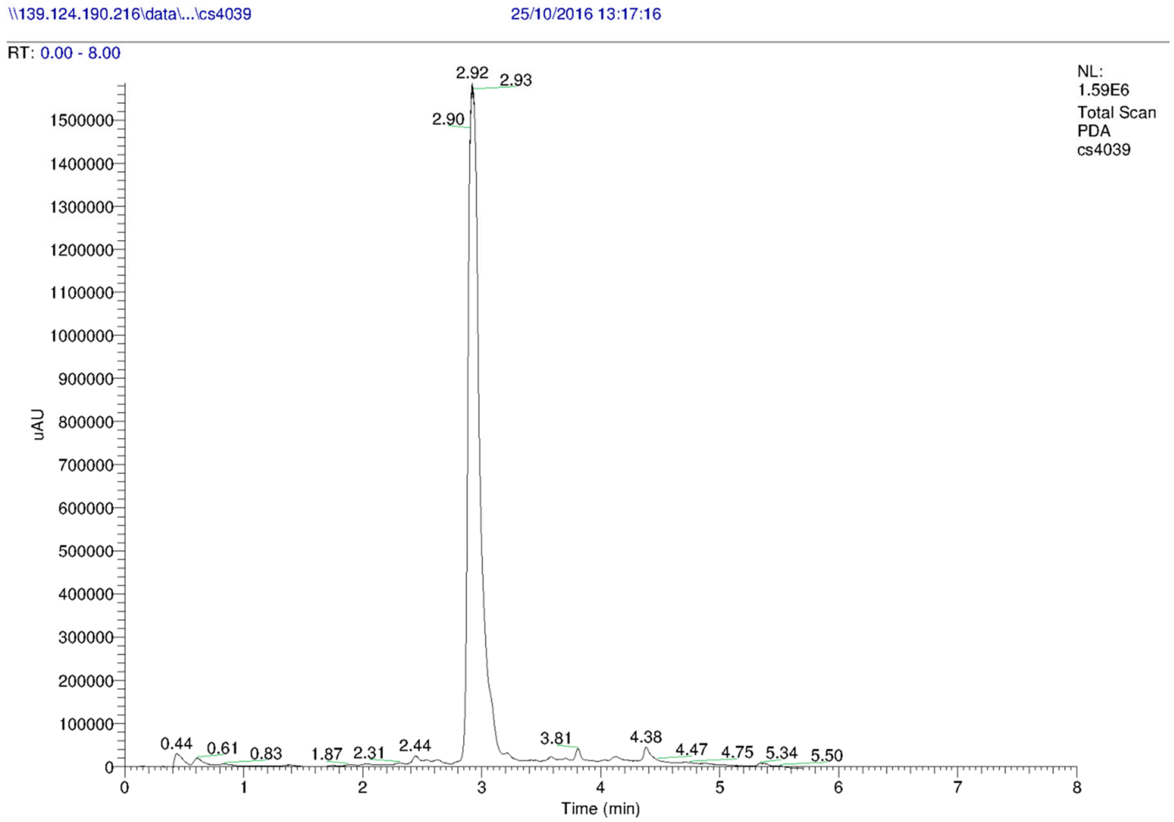

Figure S42. HPLC spectra of **6l**.
